# CD40-Activated B Cells Can Efficiently Prime Antigen-Specific Naïve CD8^+^ T Cells to Generate Effector but Not Memory T cells

**DOI:** 10.1371/journal.pone.0030139

**Published:** 2012-01-23

**Authors:** Mélissa Mathieu, Natacha Cotta-Grand, Jean-François Daudelin, Salix Boulet, Réjean Lapointe, Nathalie Labrecque

**Affiliations:** 1 Maisonneuve-Rosemont Hospital Research Center, Montréal, Québec, Canada; 2 Department of Microbiology and Immunology, University of Montreal, Montréal, Québec, Canada; 3 Centre Hospitalier de l'Université de Montréal-Hôpital Notre-Dame Research Center (CRCHUM), Montréal, Québec, Canada; 4 Department of Medicine, University of Montreal, Montréal, Québec, Canada; McGill University, Canada

## Abstract

**Background:**

The identification of the signals that should be provided by antigen-presenting cells (APCs) to induce a CD8^+^ T cell response *in vivo* is essential to improve vaccination strategies using antigen-loaded APCs. Although dendritic cells have been extensively studied, the ability of other APC types, such as B cells, to induce a CD8^+^ T cell response have not been thoroughly evaluated.

**Methodology/Principal Findings:**

In this manuscript, we have characterized the ability of CD40-activated B cells, stimulated or not with Toll-like receptor (TLR) agonists (CpG or lipopolysaccharide) to induce the response of mouse naïve CD8^+^ T cells *in vivo*. Our results show that CD40-activated B cells can directly present antigen to naïve CD8^+^ T cells to induce the generation of potent effectors able to secrete cytokines, kill target cells and control a *Listeria monocytogenes* infection. However, CD40-activated B cell immunization did not lead to the proper formation of CD8^+^ memory T cells and further maturation of CD40-activated B cells with TLR agonists did not promote the development of CD8^+^ memory T cells. Our results also suggest that inefficient generation of CD8^+^ memory T cells with CD40-activated B cell immunization is a consequence of reduced Bcl-6 expression by effectors and enhanced contraction of the CD8^+^ T cell response.

**Conclusions:**

Understanding why CD40-activated B cell immunization is defective for the generation of memory T cells and gaining new insights about signals that should be provided by APCs are key steps before translating the use of CD40-B cell for therapeutic vaccination.

## Introduction

T cells recognize via their specific T cell receptor (TCR) a peptidic fragment of the antigen (Ag) in association with MHC molecules presented at the surface of Ag-presenting cells (APCs). Following Ag encounter, T cells undergo massive proliferation and differentiate into effector T (Te) cells. After elimination of the pathogen or tumor, most Te cells die by apoptosis while a few differentiate into memory T (Tm) cells providing long term protection against re-infection or tumor relapse. The success of vaccination is dependent on the generation and long-term maintenance of functional Ag-specific Tm cells. However, little is known about the signals that should be provided by APCs to promote Tm cell development.

Efficient priming of naïve CD8^+^ T cells depends on the provision by APCs of three key signals to T cells. First, APCs should present, embedded in their major histocompatibility complex (MHC) class I molecules, a peptidic fragment of the Ag. Second, co-stimulation via CD80 and CD86 expressed by APCs is essential to induce naïve CD8^+^ T cell response. Third, inflammatory signals, such as interleukin (IL)-12 or type I interferons (IFNs), are necessary to induce an optimal response of CD8^+^ T cells [1]. Furthermore, several other molecules expressed by APCs have been shown to influence CD8^+^ T cell response. Among those, cytokines, costimulatory molecules of the tumor necrosis factor (TNF) family, Notch ligands and adhesion molecules have been shown to play a role at different stages of the T cell response. However, the exact signals that should be provided by APCs to induce efficient generation of CD8^+^ Tm cells are still unknown. This knowledge is crucial to improve vaccination strategy and to better define the type of APCs that should be used for therapeutic vaccination.

Several studies have shown that vaccination with Ag-pulsed dendritic cells (DCs) is very efficient to promote the development of functional and long-lived CD8^+^ Tm cells [2], [3]. Very interestingly, CD8^+^ Tm cell generation is accelerated with DC vaccination when compared to immunization with live pathogens [2]. This is mostly due to the low level of inflammation that promotes the formation of memory precursor effector cells (MPECs) expressing high level of CD127 and low level of KLRG1 [2]–[4]. The excellent Ag presentation capability of DCs and their powerful ability to prime naïve T cells have put these cells at the forefront of therapeutic vaccination strategies to treat cancer or infected patients. However, this approach has not been extremely successful yet. Furthermore, DCs are present in very low number in peripheral blood which limits their use [5]. Therefore, investigating the ability of other more abundant APC types, such as activated B cells, to induce a CD8^+^ T cell response might help to design better vaccination strategy and to gain knowledge about the signals that APCs should provide for the development of CD8^+^ Tm cells.

Little is known on the potential of other APCs, such as B cells, to induce the generation of Te and Tm cells. Previous studies have shown that immunization with naïve resting B cells induce T cell unresponsiveness in naïve CD4^+^ [6]–[12] and CD8^+^ T cells [11]. This tolerance induction probably results from poor expression of co-stimulatory molecules by naïve B cells. Although activation of B cells with LPS induced expression of CD80 and CD86, this was not sufficient to induce T cell priming [11], [13] indicating that other signals should be provided to B cells. More recent studies have shown that human CD40-activated B (CD40-B) cells are very good at inducing Ag-specific CD4^+^ and CD8^+^ T cell response *in vitro* [14]–[17]. Indeed, these CD40-activated B cells express high levels of co-stimulatory ligands, major histocompatibility (MHC) class I and class II molecules [14], [18], [19], CD62L and have the ability to migrate to secondary lymphoid organs [19]–[21]. These characteristics suggest that these cells might be very efficient to induce the response of naïve CD8^+^ T cells *in vivo*. In addition, the possibility to expand these cells in large number from peripheral blood [20] renders them even more attractive to use in vaccination protocol.

In this manuscript, we have characterized the ability of CD40-B cells, stimulated or not with Toll-like receptor (TLR) agonists to induce the response of naïve CD8^+^ T cells *in vivo*. Our results show that CD40-B cells can directly present Ag to naïve CD8^+^ T cells to induce the generation of potent effectors able to secrete cytokines, kill target cells and control a *Listeria monocytogenes* (Lm) infection. However, CD40-B cell immunization did not lead to the proper formation of CD8^+^ Tm cells. Although maturation of CD40-B cells with TLR agonists increased the number of Ag-specific T cells generated it did not promote the development of CD8^+^ Tm cells. Understanding why CD40-B cell immunization is defective for the generation of Tm cells and gaining new insights about signals that should be provided by APCs are key steps before translating the use of CD40-B cell for therapeutic vaccination.

## Materials and Methods

### Mice

B6SJL, C57BL/6 and OT-1 [22] mice were bred at the Maisonneuve-Rosemont Hospital Research Center facility. Bm1 mice (B6.C-H2K^bm1^/ByJ) were purchased from the Jackson Laboratory. Mice were housed in a pathogen-free environment and treated in accordance to the Canadian Council on Animal Care guidelines. Our animal protocol (number: 2007-36) was approved by the Hospital Maisonneuve-Rosemont Council on Animal Care.

### B cell and DC cultures

For B cell culture, lymphocytes were isolated on a FICOLL gradient from male C57BL/6 or B6SJL spleen followed by a 4 days culture on irradiated fibroblasts stably transfected with the CD40L cDNA (3T3-CD40L) [23] to generate CD40-B cells. Bone-marrow derived DC were generated as previously described [3]. The day before harvesting, lipopolysaccharide (LPS) (1 µg/ml) or CpG-DNA (2 mM) was added to cultures. The ovalbumin (OVA)_257–264_ peptide (SIINFEKL) or an irrelevant peptide (SIYRYYGL) (Midwest biotech) was loaded overnight on DCs (2 µg/ml) and B cells (4 µg/ml) on the last day of culture.

### Immunization and analysis of T cell response

Two days after adoptive transfer of 10^6^ OT-1 T cells (CD45.2^+^; from female mice) into female B6SJL mice (CD45.1^+^), recipients were immunized i.v. with 0.5–2×10^6^ DCs or 2–6×10^6^ CD40-B cells (from male mice to induce a CD4 T cell response against the male minor histocompatitbility antigen HY [24]) as indicated in figure legends. The presence of Te (d4 post-immunization) and Tm (d30/45 post-immunization) cells were evaluated in the same mouse by sequential removal of superficial lymph nodes as described previously [3]. The presence of Tm cells in tertiary sites and effector functions were analyzed as previously described [3]. Te and Tm cells were identified by flow cytometry as being CD8^+^ and CD45.2^+^. For immunization of Bm1 mice, 5×10^6^ CFSE-labelled OT-1 T cells were adoptively transferred in the morning and immunization were realized at least 5 h later. Mice were immunized either with 6×10^6^ CD40-B cells or 2×10^6^ DC matured with LPS and loaded or not with OVA_257–264_. OVA-specific T cells were identified using K^b^-OVA tetramer staining.

### Abs, cytometry, and cell sorting

Anti-CD86 (GL-1), -CD8 (53-6.7), -Ly6C (AL-21), CD122 (5H4) and -Bcl-2 (A19-3) were purchased from BD Biosciences. Anti-H2-K^b^ (AF6-88.5), -CD40 (IC10), -CD45.2 (104), -CD44-APC-Cy7 (1M7), -CD8 (53-6.7), CD19 (6D5), -CD11c (N418), -NK1.1 (PK136), CD27 (LG.3A10), CD80 (16-10A1), CD43 (1B11) and -IL-2 (JES6-5H4) were purchased from Biolegend. Anti- I-A^b^ (28-16-8S), -CD62L (MEL-14) and -CD11b (M1/70.15) were purchased from Caltag Laboratories. Anti-CD127 (A7R34), -Eomes-Alexa-647 (Dan11mag) and -granzyme B-PE (16G6) were purchased from eBioscience. Anti-Bcl-6-PE (7D1) and -T-bet-PE (4B10) were purchased from Santa Cruz Biotechnology. Anti-IFN-γ-FITC (XMG1.2) was purchased from Invitrogen. OVA peptide loading on K^b^ MHC was measured by staining with the 25-D1.16 Ab [25] followed by staining with a rat anti-mouse IgG1-PE (A85-1) antibody purchased from BD Bioscience. The H2K^b^-OVA monomers were purchased from CANVAC tetramer core facility and tetramers were generated using extravidin-PE. Cell surface stainings and intracellular stainings for cytokine were performed as previously described [3], [26]. Bcl-6 and Eomes intracellular stainings were performed with the FoxP3 kit from eBioscience while T-bet staining was done with the cytofix/cytoperm kit from BD Bioscience. For Bcl-2 staining, cells were stained for 30 minutes in 0.1% saponin (Sigma-Aldrich) and washed twice without saponin before cell surface staining. In some experiments, CD40-B LPS cells were sorted before immunization. Cells were stained with anti-CD11b, anti-NK1.1 and anti-CD11c Abs. Negative cells (B cells) were sorted on a FACSVantage and injected into mice.

### *In vivo* killing assay

At d4 post-immunization, equal numbers (10^7^) of B6.SJL splenocytes loaded or not with the OVA peptide and differentially stained with CFSE (250 nM or 25 nM respectively) were injected i.v. into immunized mice. 4 h later, the percentage of *in vivo* killing was determined as described previously [27]. The percentage of specific lysis was calculated as follows: ratio = (percentage CFSE low/percentage CFSE high), and percentage of specific lysis = [1−(ratio unprimed/ratio primed)]×100.

### Listeria monocytogenes (Lm) challenge

Lm was grown as previously described [28]. One lethal dose (10^5^ bacteria) of Lm expressing OVA was injected i.v. Three days post-infection, spleen and liver were harvested, homogenized, diluted and plated onto brain hearth infusion (BHI) plates containing streptomycin. After 24 h of incubation at 37°C, CFU/g of organ was determined.

### Quantitative real-time PCR

Total RNA was isolated using TRIzol (Invitrogen). RNA were reverse transcribed into cDNA using Superscript II with oligo(dT) primers (Invitrogen) as previously described [26]. Real-time PCR were performed in triplicates using SYBR Green (Invitrogen) on an Applied Biosystems 7500 Real Time PCR system. PCR was performed with mouse Blimp-1 primers: forward, 5′-ACA CAC AGG AGA GAA GCC ACA TGA-3′ and reverse, 5′TCG AAG GTG GGT CTT GAG ATT GCT-3′. The primers for the internal control hypoxanthine guanine phosphoribosyl transferase (HPRT) were: forward, 5′-CTC CTC AGA CCG CTT TTT GC-3′ and reverse, 5′-TAA CCT GGT TCA TCA TCG CTA ATC-3′ [29]. The ΔC_T_ value for each sample was determined by calculating the difference between the C_T_ value of the target and the C_T_ value of the endogenous reference gene (HPRT). Then, the ΔΔC_T_ value for each sample was determined by subtracting the mean of ΔC_T_ value of the sample from the ΔC_T_ value of a reference sample composed of cDNA from brain, thymus, spleen, activated splenocytes (anti-CD3 and anti-CD28), EL4 cell line, D011.10 cell line and SAMBOK cell line. The relative level of target gene expression was calculated using 2^−ΔΔCT^.

### Statistical analysis

Statistical analyses for differences between groups were performed using one-way ANOVA followed by Games-Howell post-tests (3 experimental groups or more) or Student's T-test (two experimental groups). Data are presented as mean +/− SEM. All tests were two-sided and p<0.05 was considered statistically significant.

## Results

### CD40-B cell immunization efficiently generates effector CD8^+^ T cells but not memory

**Figure 1:**
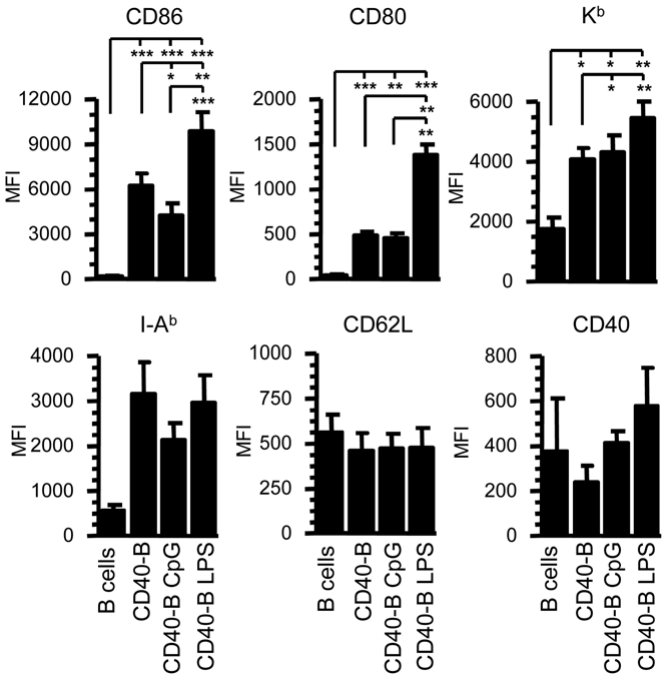
CD40-B cells have an activated phenotype. After 3 d of culture on murine 3T3-CD40L fibroblasts, CD40-B cells were matured or not with LPS (1 µg/mL) or CpG (2 mM) for 24 h (CD40-B LPS and CD40-B CpG). Freshly isolated splenocytes were used as a naïve B cell control. The histogram bars show the mean of fluorescence intensity (MFI) +/− standard deviation of the mean (SEM) for the expression of CD86, CD80, CD62L, CD40, K^b^ and I-A^b^ gated on the CD19^+^ population. The results are pooled from at least three independent experiments except for CD40 expression on B cells (n = 2). * p<0.05, ** p<0.01 and *** p<0.001.

We tested if activation of B cells with CD40L and further maturation with different TLR agonists generated APCs able to induce an *in vivo* CD8^+^ T cell response. Splenocytes were activated and expanded on fibroblasts expressing CD40L and then LPS or CpG DNA was added to stimulate TLR4 or TLR9 respectively (hereafter referred to as CD40-B LPS and CD40-B CpG). CD40 engagement on B cells resulted in up-regulation of the costimulatory molecules CD80 and CD86 but CD40-B LPS cells bore a greater activated state when compared to CD40-B and CD40-B CpG cells. (Figure 1 and Figure S1). The expression of CD62L and CD40 was similar in all conditions (Figure 1 and Figure S1). Furthermore, the expression of K^b^ and I-A^b^ MHC molecules was comparably up-regulated in the three groups of CD40-B cells (Figure 1 and Figure S1).

**Figure 2:**
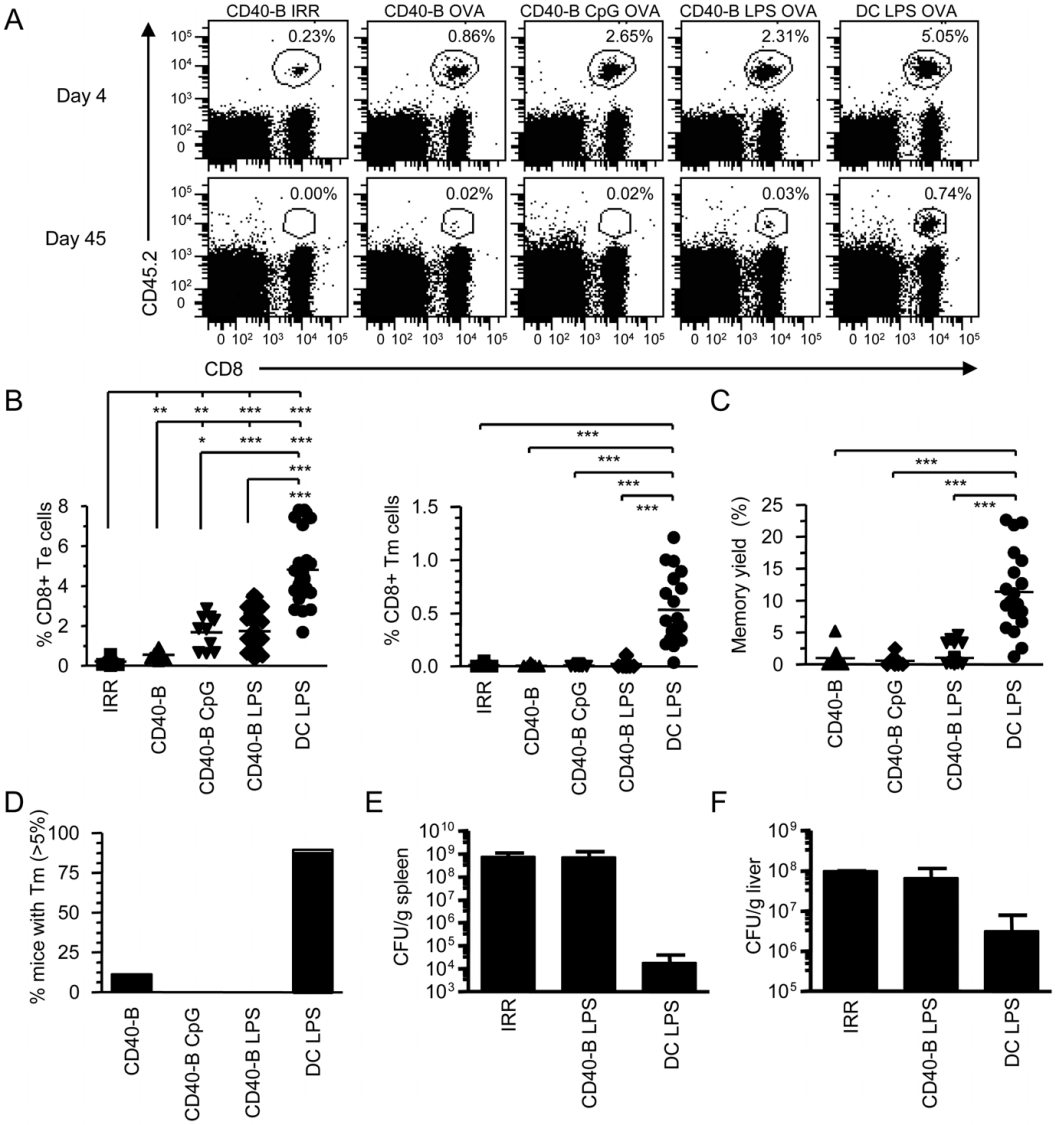
Immunization with CD40-B cells induces an *in vivo* CD8^+^ T cell response. A. CD40-B cell vaccination generates CD8^+^ Te cells but not Tm cells. 10^6^ female OT-1 T cells (CD8^+^CD45.2^+^) were adoptively transferred into congenic B6SJL female mice (CD45.1^+^) followed by immunization two days later with 2×10^6^ CD40-B cells, matured or not with LPS (1 µg/mL) or CpG (2 mM) and loaded with 4 µg/mL OVA or with an irrelevant peptide (IRR). As a reference recipients were immunized with 2×10^6^ DCs matured with LPS and loaded with OVA peptide. OVA-specific T cells (CD8^+^CD45.2^+^) were analyzed in the same mouse by surgical removal of superficial lymph nodes at d4 (effector) and d30–45 (memory) post-immunization. Te and Tm cells were identified as CD8^+^CD45.2^+^ by flow cytometry. The percentage of Te and Tm cells generated are indicated on each dot plot. B. Percentage of CD8^+^ Te (left panel) and Tm (right panel) cells recovered at d4 (Te) and d>30 (Tm) in one lymph node is shown. C. Yield of CD8^+^ Tm cell generation. The yield of Tm cell formation was calculated as the percentage of Te cells that develop into Tm cells. D. The percentage of mice that generates more then 5% of CD8^+^ Tm cells is shown for the different immunization conditions. E and F. Lm challenges. 30 d post immunization, mice were challenged with a lethal dose of Lm-OVA (10^5^ CFU). 3 d post challenged, CFU were determined in the spleen (E) and liver (F) for each mouse. A–D are from at least four independent experiments with at least two mice per group while E and F are from one independent experiment with three mice per group. * p<0.05, ** p<0.01 and *** p<0.001.

Next, we evaluated the ability of our different CD40-B cell cultures to prime CD8^+^ T cell response *in vivo*. To do so, we adoptively transferred 10^6^ naïve OVA-specific OT-1 CD8^+^ T cells followed by immunization with 2×10^6^ CD40-B, CD40-B CpG or CD40-B LPS cells pulsed with the OVA peptide. As shown in Figure S1, peptide loading was equivalent in the three groups of CD40-B cells. To provide T cell help, an endogenous anti-male response by CD4^+^ T cells was generated by immunizing female recipients, transferred with female OT-1 T cells, with male APCs. The CD8^+^ T cell response obtained after CD40-B cell immunization was always compared to the one obtained with LPS-activated DCs. Our results show that immunization with CD40-B cells allows for the generation of CD8^+^ Te cells (Figure 2A and B). However, CpG or LPS activation of CD40-B cells increases the number of Te cells (Figure 2A and B), although 2-fold less than DC immunization. We were also able to induce the response of endogenous CD8^+^ T cells (without the adoptive transfer of OT-1 T cells) to OVA using tetramer staining following CD40-B cell immunization (Figure S2).

Most strikingly, CD40-B cells matured or not with TLR ligands were almost always unable to induce CD8^+^ Tm cell development while DC immunization led to efficient CD8^+^ Tm cell generation (Figure 2A–D). Similar results were obtained in the spleen, peritoneum and bone marrow (not shown). The lack of Tm cell generation with CD40-B cell immunization is not a consequence of the reduced number of Te cells generated since Tm cells were also efficiently generated when we immunized with less DCs (Figure S3), which led to the production of less effectors. In few experiments, some CD8^+^ Tm cells were generated following CD40-B cell immunization. However, the yield of Tm cell generation was always very low (Figure 2C). None of the mice immunized with CD40-B LPS or CD40-B CpG cells developed significant number of Tm cells (at least 5% of effectors; Figure 2D) while a very low proportion of mice immunized with CD40-B cells did. These results show that TLR stimulation of CD40-B cells enhanced their *in vivo* priming potential which leads to an increased number of Ag-specific effectors but this was not sufficient to promote CD8^+^ Tm cell generation. Furthermore, we have challenged a cohort of mice where Tm cell generation was below the detection limit with Lm to confirm the lack of Tm generation. Thirty days post-immunization with CD40-B LPS cells, mice were not protected from Lm infection as shown by bacterial counts in the spleen and liver (Figure 2E and F). Notwithstanding, the few Tm cells that were generated in some experiments were functional as shown by their rapid recall response when challenged with Ag-pulsed DCs (Figure S4).

We have previously shown that the density of epitope presented by DCs critically affects the yield of CD8^+^ Tm cell generation [30]. Therefore, we have carefully compared the level of K^b^-OVA complexes at the surface of CD40-B cells and DCs. As shown, in Figure S1 and S5, CD40-B cells express a higher density of epitope than DCs. To definitively rule out that CD40-B cells were not presenting enough Ag, we have immunized mice with CD40-B cells loaded with more peptide and showed that this did not ameliorate the formation of CD8^+^ Tm cells (Figure S5).

### CD40-B cells directly present Ag to CD8^+^ T lymphocytes *in vivo*

**Figure 3:**
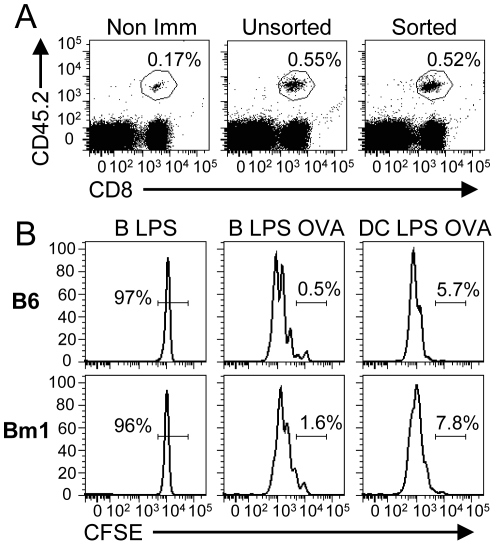
CD40-B cells directly present Ag to naïve CD8^+^ T cells. A. Highly purified sorted CD40-B cells can induce a CD8^+^ T cell response. Mice were immunized as in Figure 2 with sorted or unsorted CD40-B cells matured with LPS and loaded with OVA. Percentage of effectors (CD8^+^CD45.2^+^) generated is shown at d4 post-immunization. B. Direct priming of OVA-specific CD8^+^ T cells by CD40-B cells. 5×10^6^ CFSE-labelled OT-1 T cells were adoptively transferred into either C57BL/6 or Bm1 recipients followed by immunization on the same day with 6×10^6^ LPS-treated CD40-B cells loaded with OVA (B LPS OVA) or an irrelevant peptide (B LPS) or 2×10^6^ LPS-matured DCs loaded with OVA peptide (DC OVA). The proliferation of OVA-specific T cells was analyzed 48 h later. The histograms show CFSE-profile gated on OVA-specific CD8^+^ T cells using K^b^-OVA tetramer staining. The percentage of undivided cells is indicated on each histogram. 3 independent experiments with 2 mice per group.

At the end of the culture, we obtained an almost pure population of B cells (∼95%) with few contaminating CD11c^+^, CD11b^+^ and NK1.1^+^ cells (less than 1–2% for each population). To rule out the possibility that contaminating cells contributed to the *in vivo* priming of OT-1 T cells, we sorted B cells from the CD40-B LPS cell culture before immunization. The sorted and unsorted CD40-B LPS cells were as efficient in generating CD8^+^ Te cells at d4 post-immunization (Figure 3A). These results suggest a direct priming of CD8^+^ T cells by CD40-B LPS cells, but we could not exclude that B cells were providing the OVA peptide to endogenous APCs for cross-presentation. To rule out this possibility, we used Bm1 mice which express a variant of the MHC class I molecule H2-K^b^. The H2-K^bm1^ molecule has 3 amino acid substitutions in residues involved in peptide binding and TCR interaction rendering this molecule unable to present the OVA peptide to OT-1 T cells [31], [32]. Therefore, APCs from Bm1 mice are unable to cross-present the OVA peptide to the transferred OT-1 T cells allowing us to test if CD40-B cells can directly present the Ag and prime naïve CD8^+^ T cells. Unfortunately, this mutation is sufficient to induce rejection of transferred OT-1 T cells within 2 to 3 days [33]. To bypass this problem, we transferred into C57BL/6 or Bm1 mice 5×10^6^ CFSE labeled OT-1 T cells in the morning followed by LPS-stimulated CD40-B cell or DC immunization in the afternoon. CFSE dilution 2 d post-immunization in the spleen demonstrates that the Ag is directly presented by the injected CD40-B cells (Figure 3B). All OT-1 T cells in the Bm1 mice have proliferated, suggesting that CD40-B cells can directly present Ag to naïve CD8^+^ T cells.

### CD40-B cell or DC vaccination generates similar effectors

**Figure 4:**
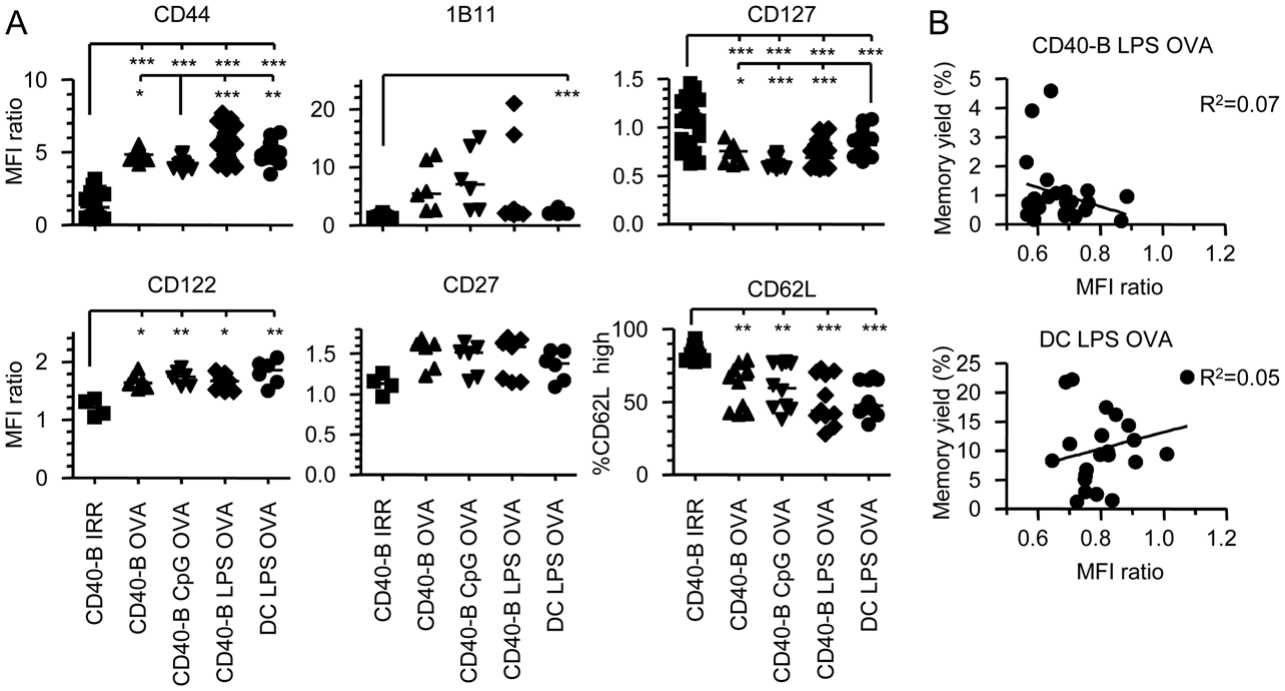
Phenotype of the CD8^+^ Te cells generated after CD40-B cell immunization. A. Phenotype of effectors at d4 post-immunization with CD40-B cells treated or not with TLR ligands. Immunizations were realized as described in Figure 2 with CD40-B cells (2×10^6^) treated or not with CpG or LPS and with DCs (2×10^6^) matured with LPS. The bar chart shows the MFI of expression for CD44, 1B11, CD127, CD122, CD27 by CD8^+^ Te cells (CD8^+^CD45.2^+^) normalized to the MFI of endogenous CD8^+^ T cells (CD8^+^CD45.2^−^). For CD62L, the percentage of CD8^+^ Te cells expressing high level of CD62L is shown. B. No correlation between CD127 expression level and memory generation. The results are from 2 independent experiments for CD27 and CD122 and from at least 3 independent experiments for the other cell surface molecules. * p<0.05, ** p<0.01 and *** p<0.001.

To understand why CD40-B cell immunization does not lead to memory generation, we have characterized Te cell phenotype. Te cells generated following CD40-B cell or DC immunization have a similar phenotype (Figure 4A and Figure S6). The level of expression of CD44, CD62L, CD27, CD122 and 1B11 were similar in the different groups indicating equivalent activation of CD8^+^ effectors following CD40-B cell or DC immunization (Figure 4A). We observed a slightly lower expression of CD127 (IL-7 receptor α chain) by effectors generated following immunization with CD40-B cells compared to DCs. Since high level of CD127 expression identifies the effectors that generate memory T cells, we have further evaluated if there was a positive correlation between the level of expression of CD127 and the yield of memory T cells. As shown in Figure 4B, there is no correlation between CD127 expression and the percentage of Tm cells generated for both CD40-B cell and DC immunizations. The lack of correlation is not surprising since more than 75% of the effectors have maintained a high level of CD127 expression (Figure S7). Therefore, it is very unlikely that the deficiency of Tm cell generation with CD40-B cell vaccination is due to lower level of CD127. In our immunization conditions, we did not obtain any increase in KLRG1 expression, a marker used to identify short-lived effectors (SLECs) in inflammatory models (not shown) [4], [34]. Due to low inflammation, most of our Te cells bear a MPEC phenotype [4]. Therefore, the cell surface phenotype of effectors generated after B cell immunization does not explain the poor generation of CD8^+^ Tm cells.

**Figure 5:**
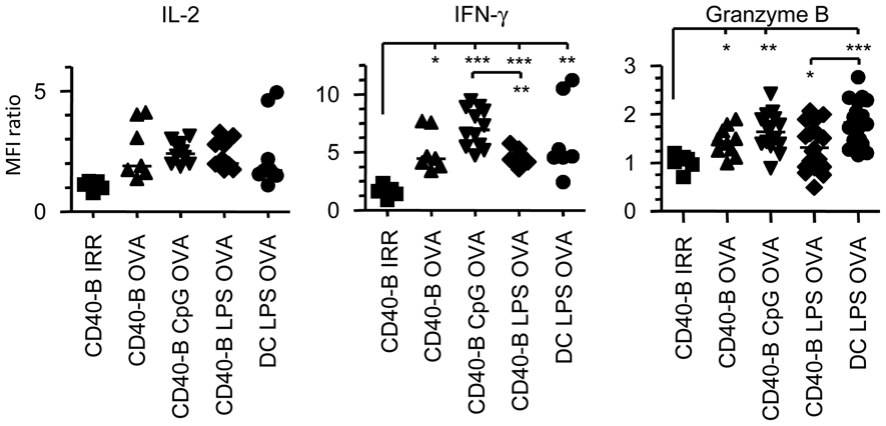
Cytokine and granzyme B production by OVA-specific CD8^+^ Te cells. Immunizations were realized as described in Figure 2 with CD40-B cells (2×10^6^–6×10^6^) treated or not with CpG or LPS and with DCs (0.5×10^5^–2×10^6^) matured with LPS. The MFI ratio was determined as in Figure 4A for IL-2, IFN-γ and granzyme B production by CD8^+^ Te cells at d4 post-immunization. Results are pooled from at least three independent experiments with a minimun of 2 mice per group. * p<0.05, ** p<0.01 and *** p<0.001.

To better characterize the potential of CD40-B cell vaccination, we evaluated effector functions of OVA-specific CD8^+^ T cells at the peak of the response. Te cells generated after B cell or DC immunization produce similar amounts of IL-2 (Figure 5 and Figure S6). All types of immunization led to IFN-γ production by effectors but CD40-B CpG cell vaccination induces higher production of IFN-γ than immunization with CD40-B LPS cells (Figure 5 and Figure S6). However, granzyme B production is lower following CD40-B cell immunization (Figure 5 and Figure S6) but this is only statistically significant for CD40-B LPS cell immunization.

### CD40-B cell immunization generates cytotoxic effectors

**Figure 6:**
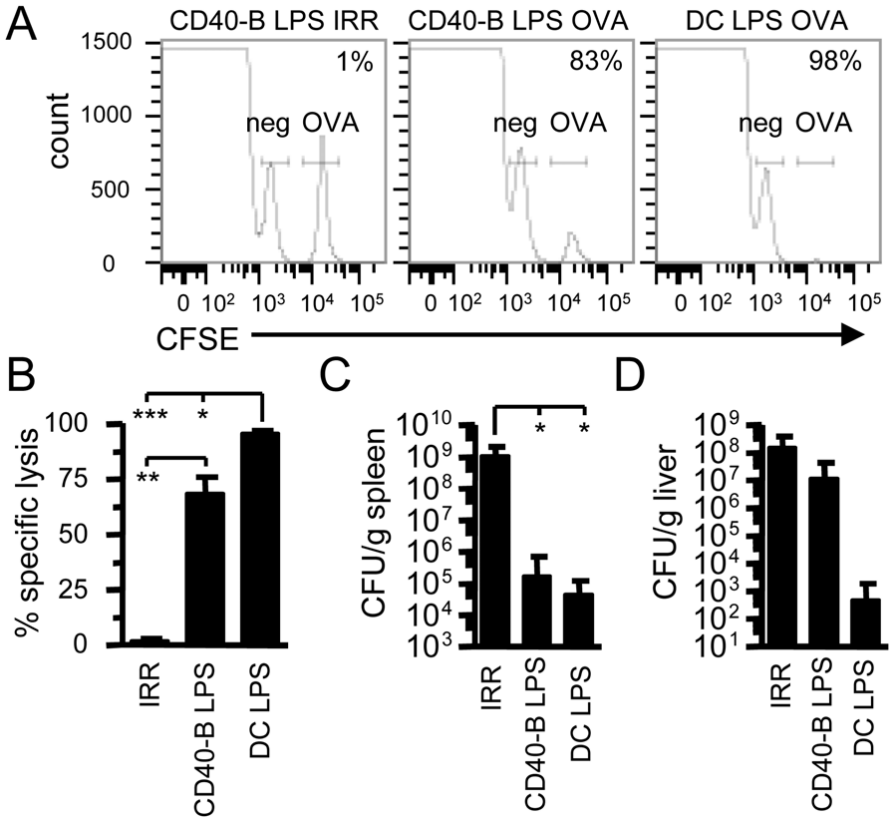
CD40-B cell vaccination generates functional effector. A. *In vivo* killing. Mice were immunized as in Figure 2. Four days post-immunization, CFSE-labeled splenocytes pulsed or not with OVA were injected as target cells. After 4 h, the percentage of CFSE^hi^ (OVA-pulsed; gate labeled OVA on the histogram) and CFSE^lo^ (unpulsed; gate labeled neg on the histogram) cells were analyzed in the spleen. Percentage of specific lysis is indicated on the histogram and was calculated using the indicated gate and as described in Material and Methods. B. Percentage of specific killing by OVA CD8^+^ Te cells. Mean +/− SEM of specific lysis are shown for the different immunization conditions. 2 mice per conditions, 3 independent experiments. C and D. Lm challenge. Four days post-immunization, mice were challenged with a lethal dose of Lm-OVA (10^5^ CFU). 3 d post-infection (peak of bacterial load), mice were killed and CFU were determined in the spleen (C) and the liver (D). Mean +/− standard (SD) are shown. 2–4 mice per conditions, 3 independent experiments. * p<0.05, ** p<0.01 and *** p<0.001.

The lower level of granzyme B production by CD8^+^ Te cells obtained after CD40-B cell vaccination led us to evaluate their ability to kill target cells *in vivo*. At d4 post-immunization, mice were injected with OVA loaded target cells and specific lysis was measured 4 h later. Te cells generated with CD40-B cell vaccination have a reduced ability to kill target cells when compared to the one obtained with DC immunization (Figure 6 A and B). The reduced cytotoxic activity of Te cells generated after CD40-B cell immunization could be a result of their lower frequency when compared to DC immunization (Figure 2). Thus, CD40-B cell immunization generates functional cytolytic effectors.

### Effectors are able to control a bacterial infection

To further assess the functionality of Te cells following CD40-B cell vaccination, we have measured their ability to control a Lm infection. Four days post-immunization, mice were challenged with one lethal dose of virulent Lm expressing OVA (Lm-OVA). Immunization with both CD40-B LPS cells or DCs led to a 4 log decrease in the spleen bacterial burden at d3 post-infection (Figure 6C). Effectors generated with DC vaccination seem better then those obtained with CD40-B cells to control Lm spreading in the liver (Figure 6D). Again, we cannot rule out the possibility that this is caused by the lower number of Te cells produced with CD40-B cell immunization. Furthermore, both types of Te cells expanded to the same extent following infection with Lm-OVA indicating that CD40-B cells do not induce T cell tolerance (Figure S8).

### Enhanced contraction and reduced Bcl-6 expression by effectors following CD40-B cell immunization

**Figure 7:**
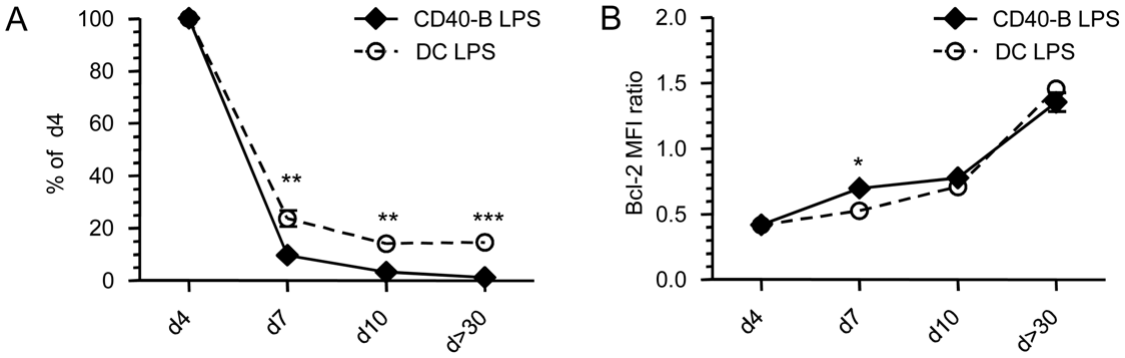
Effectors generated with CD40-B cell immunization contract more rapidly than the one obtained with DC immunization. A. Contraction of the OVA-specific CD8^+^ T cell response. Mice were immunized as described in Figure 2. Lymph nodes were surgically removed at 4, 7, 10 and >30 days post-immunization. Cells were stained to determine the percentage of Te cells generated. The graph shows the percentage of remaining Te cells (CD8^+^CD45.2^+^) over time relative to the peak of the response (d4). B. Effectors generated with CD40-B cell and DC immunization express similar amount of Bcl-2 during the course of the CD8^+^ T cell response. The MFI of Bcl-2 for OVA-specific CD8^+^ Te cells was normalized to the MFI of endogenous CD8^+^ T cells to obtain a MFI ratio. 3 independent experiments. * p<0.05, ** p<0.01 and *** p<0.001.

Our results clearly show that both DC and CD40-B cell immunizations generate fully functional CD8^+^ effectors and that only Tm cell generation is impaired following CD40-B cell vaccination. To further address why effectors generated with CD40-B cell immunization did not differentiate into Tm cells, we have evaluated the extent of T cell contraction. As shown in Figure 7A, CD8^+^ Te cells generated with CD40-B cell immunization have a higher rate of contraction than the one generated with DC immunization. Moreover, at day 10 post-immunization, CD8^+^ Te cells obtained with CD40-B cells were already almost undetectable (Figure 7A). The enhanced contraction of effectors generated with CD40-B cell immunization is not due to a lower expression of Bcl-2 (Figure 7B).

**Figure 8:**
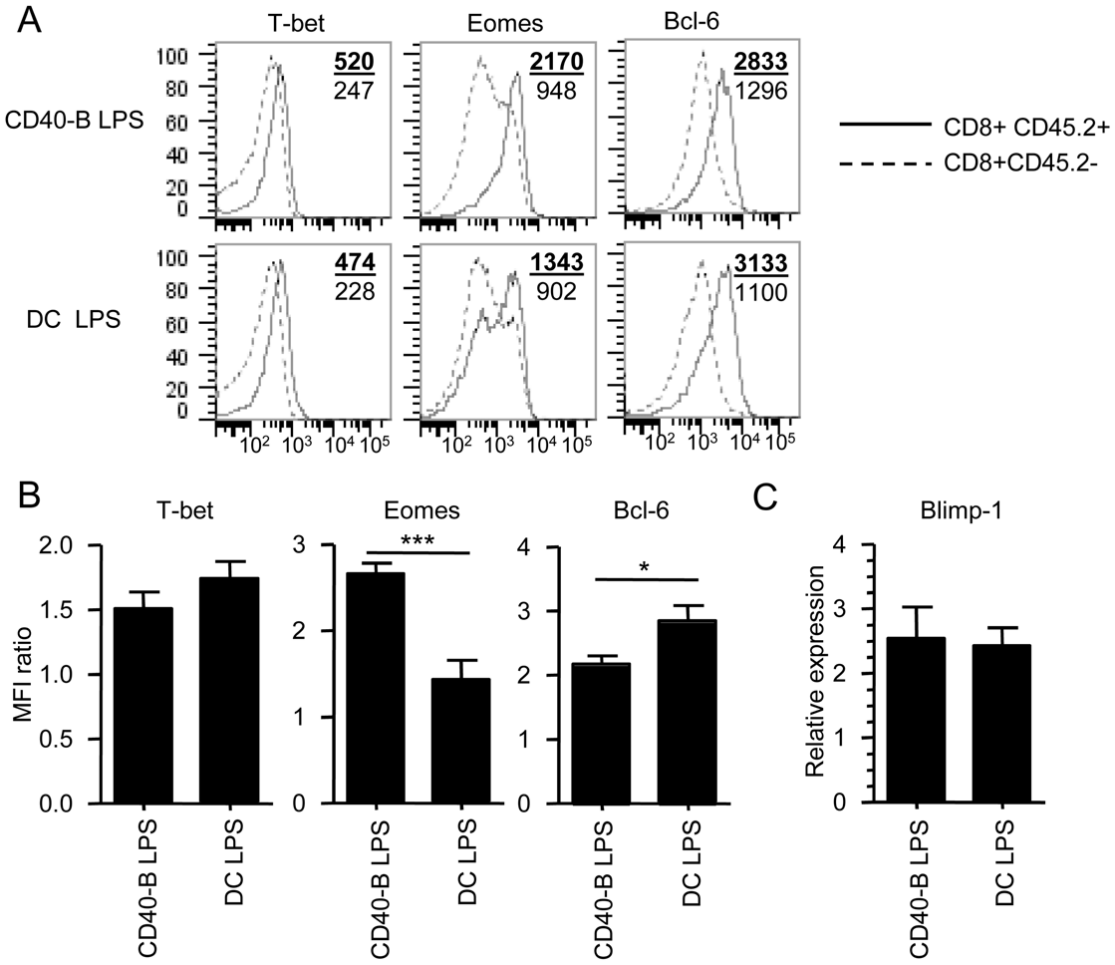
CD40-B cell immunization generates effectors expressing similar level of T-bet and Blimp-1, higher level of Eomes and lower amount of Bcl-6. A. Expression of T-bet, Eomes and Bcl-6 by CD8^+^ Te cells generated following CD40-B cell and DC immunizations. Four days post-immunization with 2×10^6^ CD40-B cells or DCs matured with LPS and loaded with the OVA peptide, Te cells were stained intracellularily with antibodies against T-bet, Eomes and Bcl-6 transcription factors. The representative overlay histogram shows expression of the transcription factor by endogenous T cells (CD8^+^CD45.2^−^) and OVA-specific Te cells (CD8^+^CD45.2^+^). The MFI is shown on each overlay, the upper bold number indicates the MFI of OVA-specific effectors (CD8^+^CD45.2^+^) while the lower number is for the endogenous population (CD8^+^CD45.2^−^). B. Quantification of the level of expression of T-bet, Eomes and Bcl-6. The histograms shows the MFI of expression for T-bet, Eomes and Bcl-6 by OVA-specific CD8^+^ Te cells (CD8^+^CD45.2^+^) normalized to the MFI of endogenous CD8^+^ T cells (CD8^+^CD45.2^−^). The results are from at least 2 independent experiments. C. Similar expression of Blimp-1 by OVA-specific Te cells following CD40-B cell or DC immunization. At the peak of the response (d4), Te cells were sorted (CD8^+^CD45.2^+^) from spleen to extract RNA. The relative expression of Blimp-1 was determined by quantitative RT-PCR. Expression relative to a reference sample is shown. Results are from 4 independent experiments. * p<0.05 and *** p<0.001.

The transcriptional network controlling CD8^+^ Tm cell generation has been partially elucidated over the last years. During CD8^+^ T cell response to infection, the effectors (short-lived effectors or SLECs) that will die by apoptosis expressed high level of the transcription factor T-bet while MPECs express lower amount of T-bet [4]. The level of expression of the transcription factor Eomes inversely correlates with the level of T-bet suggesting that high level of Eomes are necessary to promote MPEC development and Tm cell formation [29], [35], [36]. Furthermore, Eomes expression is needed for CD8^+^ Tm cell self-renewal [37]. Similarly to T-bet, the transcriptional repressor Blimp-1 controls the formation of SLECs and in its absence MPECs are preferentially generated [38], [39]. The transcriptional repressor Bcl-6 is required for CD8^+^ Tm cell generation but unlike T-bet, Eomes and Blimp-1 it does not contribute to effector formation [40], [41]. Furthermore, Blimp-1 represses Bcl-6 expression allowing for the differentiation of SLECs. Therefore, the balance of expression of T-bet, Blimp-1, Eomes and Bcl-6 is crucial for efficient development of CD8^+^ Tm cells. To elucidate why CD40-B cell immunization is not efficient to generate CD8^+^ Tm cells, we evaluated the expression level of these key transcription factors in Te cells (CD8^+^CD45.2^+^) at the peak of the response (d4 post-immunization). We could not detect any variation in T-bet and Blimp-1 expression in Te cells generated with CD40-B LPS or DC immunization (Figure 8). Moreover, effectors generated with CD40-B cell immunization express higher amount of Eomes (Figure 8), which should be beneficial for CD8^+^ Tm cell generation and self-renewal. These results suggest that CD8^+^ Te cells that are generated with CD40-B cell immunization are not more terminally differentiated than the ones obtained with DC immunization. However, we constantly observed a 25% reduction in the level of expression of Bcl-6 in effectors obtained after CD40-B cell immunization when compared to DC vaccination (Figure 8). This suggests that CD40-B cells are unable to promote high level of expression of Bcl-6, a key transcription factor involved in the generation of CD8^+^ Tm cells, and that this may leads to inefficient differentiation of Te cells into memory cells.

## Discussion

To better understand the signals given by different APCs, we have compared CD8^+^ T cell responses generated after CD40-B cell or DC immunization. Our results clearly demonstrate that CD40-B cells can present Ag and activate naïve CD8^+^ T cells *in vivo*. It is not surprising that CD40-B cells can act as efficient APCs *in vivo* since CD40 stimulation leads to the up-regulation of MHC class I and II molecules, CD80 and CD86 co-stimulatory molecules allowing CD40-B cells to present Ag and provide efficient co-stimulation to naïve CD8^+^ T cells. Previous reports have shown that B cells are tolerogenic APCs [6]–[12] but they used small resting B cells lacking CD80 and CD86 expression, thus explaining why no priming occurs. In our study, we have also investigated if CD40-B cells directly present Ag to CD8^+^ T cells or if they only provide Ag for cross-presentation by endogenous APCs. Using Bm1 mice as recipients for vaccination, we clearly show that CD40-B cell directly act as APCs and that cross-presentation by DCs is not necessary to induce a CD8^+^ T cell response. Therefore, CD40-B cells are good APCs to prime naïve CD8^+^ T cells *in vivo* opening the potential for their use in vaccination.

When compared to DC, CD40-B cell vaccination generates 2 fold less effectors even after maturation with LPS or CpG. This small difference is not a clinical issue since it could be easily compensated by increasing the number of CD40-B cells used in immunization. In fact, we have observed a similar expansion of Ag-specific CD8^+^ T cells when we used 3–4 times more CD40-B cells than DCs (Figure S3). Increasing the number of CD40-B cells in immunization is a relevant solution because it is very easy to generate them in large numbers from small amounts of human blood. Therefore, our results suggest that CD40-B cell immunization can be as good as DC immunization to generate Ag-specific CD8^+^ Te cells. Our results also show that further maturation of CD40-B cells with TLR agonists, CpG or LPS, increases the number of Ag-specific CD8^+^ T cells generated. Therefore, in vaccination protocols, it would be advantageous to activate CD40-B cells with TLR agonists to increase effector generation.

Our results also show that CD40-B cell vaccination is able to induce the proper differentiation of naïve CD8^+^ T cells into functional effectors. Only a minor difference was observed at the functional level, effectors obtained with CD40-B LPS cell immunization produce less granzyme B than the one obtained with DC vaccination. Although producing less granzyme B, these effectors were able to kill target cells *in vivo*. This indicates that CD40-B cell vaccination is able to provide all the necessary signals to generate cytolytic effectors. Furthermore, effectors generated with CD40-B cell immunization expressed similar level of IFN-γ and IL-2 when compared to Ag-specific CD8^+^ T cells obtained with DC immunization. The production of IFN-γ by effectors obtained with CD40-B cell vaccination is very interesting since IFN-γ production correlates positively with clinically effective anti-tumor response [42]–[52]. Moreover, Te cells generated with CD40-B cell vaccination are able to control a Lm infection. Thus, our results show that CD40-B cell immunization generates functional effectors able to produce IFN-γ, to kill target cells and to control bacterial infection. Therefore, the ability of CD40-B cell vaccination to produce potent effectors renders this approach of interest to induce anti-tumor T cell response in cancer patients.

It is intriguing that CD40-B cell vaccination does not generate T cell memory since most of the Te cells generated have a MPEC phenotype (CD127^hi^KLRG1^lo^). The deficient Tm cell generation is not due to the reduced number of effectors since decreasing the number of Te cells generated with DC immunization still lead to the formation of Tm cells (Figure S3). Therefore, the deficient Tm cell generation suggests that priming with CD40-B cells does not provide all the necessary signals to allow the differentiation of naïve CD8^+^ T cells into long-lived memory T cells. Further investigations are needed to understand what is missing in CD40-B cells.

To better understand why CD40-B cell vaccination is not efficient in promoting CD8^+^ Tm cell generation, we evaluated the expression of T-bet, Eomes, Blimp-1 and Bcl-6 which are key transcription factors known to influence memory generation and maintenance [4], [29], [35]–[39]. No differences were observed in the expression of T-bet and Blimp-1 in Te cells obtained from CD40-B cell and DC immunizations. Since the expression level of these transcription factors control the fate between SLECs and MPECs [29], [35], [36], [38], [39], [53], our results suggest that Te cells generated with CD40-B cell immunization are not more terminally differentiated than the ones obtained with DC immunization and that defective expression of these transcription factors are not responsible for the poor memory generation that we observed with CD40-B cell vaccination. Although effectors generated following CD40-B cell immunization express higher level of Eomes they are not able to differentiate into Tm cells suggesting that high level of Eomes is not sufficient to promote CD8^+^ Tm cell development. However, Te cells generated with CD40-B cell immunization express lower amount of the transcriptional repressor Bcl-6, known to control CD8^+^ Tm cell development [40], [41], than effectors generated with DC immunization. Therefore, it is possible that this small (25%) reduction in Bcl-6 expression is responsible for the poor development of CD8^+^ Tm cells following CD40-B cell vaccination. Further studies should reveal whether reduced Bcl-6 expression is solely responsible for the lack of CD8^+^ Tm cell generation or if improper induction of other transcription factors contributes to defective formation of CD8^+^ Tm cells with CD40-B cell immunization.

In our hands, no protection was achieved when mice were challenged with Lm at d30 post-immunization. This is in contrast with the results of Vanden Bush et al [54] reporting successful protection from Lm infection 30 d after CD40-B cell vaccination. However, in their manuscript, they only look at protection and did not enumerate the number of CD8^+^ Tm cells generated. It is possible that a very low number of CD8^+^ Tm cells are generated with CD40-B cell vaccination rendering their detection difficult. However, it is very clear from our results that in some experiments, but not all, CD8^+^ Tm cells are generated and are able to respond to a recall immunization with DCs. Therefore, it is possible that mice will be protected from a Lm challenge when few CD8^+^ Tm cells are produced. The difference might also be explained by the protocol used to generate CD40-B cells. Indeed, Bishop's team used CD40-B cells that spent only one day in culture [54] while we used cells cultured for 4 days. We believe that our protocol is closer to what is currently developed for human B cell expansion [15], [20]. Thus, it will be primordial to determine if the culture regimen impacts the ability of CD40-B cells to activate the immune system.

Although CD8^+^ Tm cell generation is not optimal with CD40-B cell vaccination such an approach might be viable in vaccination strategy if a boosting regimen is included. Indeed, our preliminary results suggest that it is possible to further re-expand the few Tm cells that were generated in some experiments.

Understanding why CD40-B cell immunization does not promote efficient CD8^+^ Tm cell differentiation will help our understanding of the mechanisms regulating CD8^+^ Tm cell generation and should allow for improvement of the APC potential of CD40-B cells. Furthermore, the ability of CD40-B cell vaccination to generate cytolytic effectors producing IFN-γ might be very beneficial for therapeutic vaccination.

## Supporting Information

Figure S1 **Characterization of CD40-B cells.** A. Phenotype of CD40-B cells and DCs. B cells were grown from B6SJL splenocytes on irradiated murine NIH-3T3 fibroblasts transfected with the mouse CD40L cDNA. After 3 days of culture, B cells were matured with LPS (1 µg/mL) or CpG-DNA (2 mM) for 24 h. Freshly isolated splenocytes were also stained as controls. Dendritic cells (DC) were obtained by culturing bone marrow cells during 7 days with GM-CSF and IL-4 and were matured with LPS (1 µg/mL) on day 6. Percentage of CD19^+^ cells, percentage of CD11c^+^ cells or mean fluorescence intensity are indicated on each histogram. CD86, CD80, IA^b^, K^b^, and CD62L histograms were gated on CD19^+^ cells for CD40-B cells and splenocytes or on CD11c^+^ cells for DCs. B. Similar loading of the OVA peptide for each B cell culture conditions. SIINFEKL (OVA) peptide (4 µg/ml) was added on day 3 of culture. The Kb-OVA complex was detected using a monoclonal antibody (25.D1.16) that specifically recognizes this peptide-MHC complex. The staining for K^b^-OVA is shown for each culture conditions.
(TIF)

Figure S2 **Immunization with CD40-B lymphocytes induces an endogenous response.** C57BL/6 mice were immunized with 2×10^6^ CD40-B cells or DCs matured with LPS (1 µg/mL) and loaded with SIINFEKL (OVA) peptide. Responses were measured in the blood 7 days post-immunization with K^b^-OVA tetramer staining. A. Representative dot plot are shown. Cells were previously gated on CD8^+^CD3^+^ cells. B. Percentage of response for each immunized mouse is shown. 2 independent experiments. Statistical analysis was done using a t-test. *p<0.05 and **p<0.01.
(TIF)

Figure S3 **Immunization with CD40-B cells can generate an effector response comparable to immunization with DCs.** 10^6^ female OT-1 cells were adoptively transferred into B6SJL female mice followed by immunization with 2×10^6^ or 6×10^6^ CD40-B cells, matured with LPS (1 µg/mL) or CpG-DNA (2 mM) and loaded with 4 µg/mL of SIINFEKL (OVA) peptide. As a reference recipients were immunized with 0.5×10^6^ or 2×10^6^ DCs matured with LPS and loaded with OVA peptide. OVA-specific T cells (CD8^+^CD45.2^+^) effector response was analyzed in the same mouse by surgical removal of superficial lymph nodes at d4 and d30 or more. A. Percentage of Te cells (CD8^+^CD45.2^+^) generated with 6×10^6^ (6 M) CD40-B LPS OVA, 6 M CD40-B CpG OVA or 2×10^6^ (2 M) DC LPS OVA immunization. B. Percentage of Te cells (CD8^+^CD45.2^+^) obtained with 2 M CD40-B LPS OVA, 2 M CD40-B CpG OVA or 0.5×10^6^ (0.5 M) DC LPS OVA immunization. C. Percentage of Tm cells (CD8^+^CD45.2^+^) generated following immunization with 2 M CD40-C CpG OVA, 2 M CD40-B LPS OVA or 0.5 M DC LPS OVA. D. Memory yield (% of Tm cells generated from Te cells) is shown for the different immunization conditions (as in B). Each dot represents one mouse. Medians are shown and Kruskall-Wallis statistical analysis was performed. ** p<0.01, *** P<0.001.
(TIF)

Figure S4 **Recall response of CD8^+^ memory T cells after a challenge with Ag-pulsed DCs.** 10^6^ female OT-1 T cells (CD8^+^CD45.2^+^) were adoptively transferred into congenic B6SJL female mice (CD45.1^+^) followed by immunization two days later with 2×10^6^ CD40-B cells, matured or not with LPS (1 µg/mL) or CpG (2 mM) and loaded with 4 ug/mL of OVA peptide or with an irrelevant peptide (IRR). As a reference recipients were immunized with 2×10^6^ DCs matured with LPS and loaded with OVA peptide. The presence of OVA-specific T cells (CD8^+^CD45.2^+^) were analyzed in the same mouse by surgical removal of superficial lymph nodes at d4 (effector; top row) and d44 (memory; middle row) of the primary response. At d45 post-immunisation, mice were challenged or not with 1.25×10^6^ DCs matured with LPS and pulsed with the OVA peptide. Three days (d48) post-challenge, mice were sacrificed and OVA specific CD8^+^ memory T cell (CD8^+^CD45.2^+^) expansion was evaluated in the lymph nodes (bottom row).
(TIF)

Figure S5 **Defective memory T cell generation with CD40-B cell vaccination is not due to lower epitope density.** A. CD40-B cells express a higher density of epitope than DCs. The level of K^b^-OVA MHC-peptide complex expressed by the different APCs was evaluated using a monoclonal antibody (25.D1.16) that specifically recognizes this peptide-MHC complex. The staining for K^b^-OVA is shown for each APC type used for immunization. The mean fluorescence intensity of K^b^-OVA expression is indicated for each APC type next to the legend. B. Increasing epitope density on CD40-B cells does not improve the generation of CD8^+^ memory T cells. 10^6^ female OT-1 T cells (CD8^+^CD45.2^+^) were adoptively transferred into congenic B6.SJL female mice (CD45.1^+^) followed by immunization two days later with 2×10^6^ CD40-B cells, matured with LPS (1 µg/mL) and loaded with different concentrations of OVA peptide (4 or 10 µg/ml) or with an irrelevant peptide (IRR). As a reference recipients were immunized with 2×10^6^ DCs matured with LPS and loaded with 2 µg/ml of OVA peptide. The dot plots show the effector (d4) and memory responses (d32) for each vaccination conditions. The percentage of effector and memory (CD8^+^CD45.2^+^) T cells generated are indicated on each dot plot.
(TIF)

Figure S6 **Phenotype and function of effector CD8^+^ T cells generated after CD40-B cell immunization.** A. Phenotype of OVA-specific CD8^+^ effector T cells. 10^6^ female OT-1 cells were adoptively transferred into B6SJL female mice followed by immunization with 2×10^6^ CD40-B cells, matured or not with LPS (1 µg/mL) or CpG-DNA (2 mM) and loaded with 4 µg/mL SIINFEKL (OVA) or an irrelevant peptide (IRR). As a reference recipients were immunized with 2×10^6^ DCs matured with LPS and loaded with OVA peptide. 4 days post-immunization, lymph nodes were removed by surgery and the phenotype of effectors was analysed by flow cytometry. The overlays show expression of the different cell surface markers by OVA-specific T cells (CD8^+^CD45.2^+^) compared to endogenous T cells (CD8^+^CD45.2^−^). The mean fluorescence intensity (MFI) is indicated on each histogram, the upper bold number indicates the MFI of OVA-specific effectors (CD8^+^CD45.2^+^) while the lower number is for the endogenous population (CD8^+^CD45.2^−^). B. Acquisition of effector functions by OVA-specific CD8^+^ T cells following CD40-B cell vaccination. OVA-specific effector CD8^+^ T cells were generated as in A. 4 days post-immunization, cells were restimulated for 4 h with PMA/ionomycin in the presence of brefeldin A to evaluate IFN-γ, IL-2 and granzyme B production. The overlays show production of the different effector molecules by OVA-specific T cells (CD8^+^CD45.2^+^) compared to endogenous T cells (CD8^+^CD45.2^−^). The MFI is indicated on each histogram, the upper bold number indicates the MFI of OVA-specific effectors (CD8^+^CD45.2^+^) while the lower number is for the endogenous population (CD8^+^CD45.2^−^).
(TIF)

Figure S7 **The majority of effectors generated with CD40-B cell immunization express high level of CD127.** 10^6^ female OT-1 cells were adoptively transferred into B6SJL female mice followed by immunization with 2×10^6^ CD40-B cells or DCs matured with LPS (1 µg/mL) and loaded with SIINFEKL (OVA). OVA-specific T cells (CD8^+^CD45.2^+^) effectors response was analyzed in superficial lymph nodes at d4 post-immunization. Percentage of effectors highly expressing CD127 are shown. 3 independent experiments, 2–3 mouse per experiments.
(TIF)

Figure S8 **Similar CD8+ effector T cell expansion following Listeria monocytogenes challenge.** 10^6^ female OT-1 T cells (CD8^+^CD45.2^+^) were adoptively transferred into congenic B6SJL female mice (CD45.1^+^) followed by immunization two days later with 2×10^6^ CD40-B cells, matured or not with LPS (1 µg/mL) and loaded with 4 µg/mL OVA or with an irrelevant peptide (IRR). As a reference recipients were immunized with 5×10^5^ DCs matured with LPS and loaded with OVA peptide. Four days post-immunization, mice were challenged with a lethal dose of Lm-OVA (10^5^ CFU). On the day of challenge and 3 d post-infection (peak of bacterial load) blood was harvested, stained and analyzed by flow cytometry. Percentage of CD8^+^CD45.2^+^ effectors before and after challenge were determined to calculate fold expansion. Mean +/− SEM are shown. 2–4 mice per conditions, 3 independent experiments. * p<0.05 and ** p<0.01.
(TIF)

## References

[1] Mescher MF, Curtsinger JM, Agarwal P, Casey KA, Gerner M (2006). Signals required for programming effector and memory development by CD8+ T cells.. Immunol Rev.

[2] Badovinac VP, Messingham KA, Jabbari A, Haring JS, Harty JT (2005). Accelerated CD8+ T-cell memory and prime-boost response after dendritic-cell vaccination.. Nat Med.

[3] Lacombe MH, Hardy MP, Rooney J, Labrecque N (2005). IL-7 receptor expression levels do not identify CD8+ memory T lymphocyte precursors following peptide immunization.. J Immunol.

[4] Joshi NS, Cui W, Chandele A, Lee HK, Urso DR (2007). Inflammation directs memory precursor and short-lived effector CD8(+) T cell fates via the graded expression of T-bet transcription factor.. Immunity.

[5] Lopez JA, Bioley G, Turtle CJ, Pinzon-Charry A, Ho CS (2003). Single step enrichment of blood dendritic cells by positive immunoselection.. J Immunol Methods.

[6] Eynon EE, Parker DC (1992). Small B cells as antigen-presenting cells in the induction of tolerance to soluble protein antigens.. J Exp Med.

[7] Lassila O, Vainio O, Matzinger P (1988). Can B cells turn on virgin T cells?. Nature.

[8] Ronchese F, Hausmann B (1993). B lymphocytes in vivo fail to prime naive T cells but can stimulate antigen-experienced T lymphocytes.. J Exp Med.

[9] Raimondi G, Zanoni I, Citterio S, Ricciardi-Castagnoli P, Granucci F (2006). Induction of peripheral T cell tolerance by antigen-presenting B cells. I. Relevance of antigen presentation persistence.. J Immunol.

[10] Raimondi G, Zanoni I, Citterio S, Ricciardi-Castagnoli P, Granucci F (2006). Induction of peripheral T cell tolerance by antigen-presenting B cells. II. Chronic antigen presentation overrules antigen-presenting B cell activation.. J Immunol.

[11] Fuchs EJ, Matzinger P (1992). B cells turn off virgin but not memory T cells.. Science.

[12] Gilbert KM, Weigle WO (1994). Tolerogenicity of resting and activated B cells.. J Exp Med.

[13] Parekh VV, Prasad DV, Banerjee PP, Joshi BN, Kumar A (2003). B cells activated by lipopolysaccharide, but not by anti-Ig and anti-CD40 antibody, induce anergy in CD8+ T cells: role of TGF-beta 1.. J Immunol.

[14] Lapointe R, Bellemare-Pelletier A, Housseau F, Thibodeau J, Hwu P (2003). CD40-stimulated B lymphocytes pulsed with tumor antigens are effective antigen-presenting cells that can generate specific T cells.. Cancer Res.

[15] Schultze JL, Michalak S, Seamon MJ, Dranoff G, Jung K (1997). CD40-activated human B cells: an alternative source of highly efficient antigen presenting cells to generate autologous antigen-specific T cells for adoptive immunotherapy.. J Clin Invest.

[16] von Bergwelt-Baildon MS, Vonderheide RH, Maecker B, Hirano N, Anderson KS (2002). Human primary and memory cytotoxic T lymphocyte responses are efficiently induced by means of CD40-activated B cells as antigen-presenting cells: potential for clinical application.. Blood.

[17] Coughlin CM, Vance BA, Grupp SA, Vonderheide RH (2004). RNA-transfected CD40-activated B cells induce functional T-cell responses against viral and tumor antigen targets: implications for pediatric immunotherapy.. Blood.

[18] Shen S, Xu Z, Qian X, Ding Y, Yu L (2007). Autogeneic rna-electroporated CD40-ligand activated b-cells from hepatocellular carcinoma patients induce CD8+ T-cell responses ex vivo.. Exp Oncol.

[19] Ahmadi T, Flies A, Efebera Y, Sherr DH (2008). CD40 Ligand-activated, antigen-specific B cells are comparable to mature dendritic cells in presenting protein antigens and major histocompatibility complex class I- and class II-binding peptides.. Immunology.

[20] Kondo E, Gryschok L, Klein-Gonzalez N, Rademacher S, Weihrauch MR (2009). CD40-activated B cells can be generated in high number and purity in cancer patients: analysis of immunogenicity and homing potential.. Clin Exp Immunol.

[21] von Bergwelt-Baildon M, Shimabukuro-Vornhagen A, Popov A, Klein-Gonzalez N, Fiore F (2006). CD40-activated B cells express full lymph node homing triad and induce T-cell chemotaxis: potential as cellular adjuvants.. Blood.

[22] Hogquist KA, Jameson SC, Heath WR, Howard JL, Bevan MJ (1994). T cell receptor antagonist peptides induce positive selection.. Cell.

[23] Kershaw MH, Hsu C, Mondesire W, Parker LL, Wang G (2001). Immunization against endogenous retroviral tumor-associated antigens.. Cancer Res.

[24] Livingstone AM, Kuhn M (1999). Dendritic cells need T cell help to prime cytotoxic T cell responses to strong antigens.. Eur J Immunol.

[25] Porgador A, Yewdell JW, Deng Y, Bennink JR, Germain RN (1997). Localization, quantitation, and in situ detection of specific peptide-MHC class I complexes using a monoclonal antibody.. Immunity.

[26] Ostiguy V, Allard EL, Marquis M, Leignadier J, Labrecque N (2007). IL-21 promotes T lymphocyte survival by activating the phosphatidylinositol-3 kinase signaling cascade.. J Leukoc Biol.

[27] Coles RM, Mueller SN, Heath WR, Carbone FR, Brooks AG (2002). Progression of armed CTL from draining lymph node to spleen shortly after localized infection with herpes simplex virus 1.. J Immunol.

[28] Bahjat KS, Liu W, Lemmens EE, Schoenberger SP, Portnoy DA (2006). Cytosolic entry controls CD8+-T-cell potency during bacterial infection.. Infect Immun.

[29] Intlekofer AM, Takemoto N, Wherry EJ, Longworth SA, Northrup JT (2005). Effector and memory CD8+ T cell fate coupled by T-bet and eomesodermin.. Nat Immunol.

[30] Leignadier J, Labrecque N (2010). Epitope density influences CD8 memory T cell differentiation.. PLoS One.

[31] Schulze DH, Pease LR, Geier SS, Reyes AA, Sarmiento LA (1983). Comparison of the cloned H-2Kbm1 variant gene with the H-2Kb gene shows a cluster of seven nucleotide differences.. Proc Natl Acad Sci U S A.

[32] Bjorkman PJ, Saper MA, Samraoui B, Bennett WS, Strominger JL (1987). The foreign antigen binding site and T cell recognition regions of class I histocompatibility antigens.. Nature.

[33] Loyer V, Fontaine P, Pion S, Hetu F, Roy DC (1999). The in vivo fate of APCs displaying minor H antigen and/or MHC differences is regulated by CTLs specific for immunodominant class I-associated epitopes.. J Immunol.

[34] Hand TW, Morre M, Kaech SM (2007). Expression of IL-7 receptor alpha is necessary but not sufficient for the formation of memory CD8 T cells during viral infection.. Proc Natl Acad Sci U S A.

[35] Intlekofer AM, Takemoto N, Kao C, Banerjee A, Schambach F (2007). Requirement for T-bet in the aberrant differentiation of unhelped memory CD8+ T cells.. J Exp Med.

[36] Takemoto N, Intlekofer AM, Northrup JT, Wherry EJ, Reiner SL (2006). Cutting Edge: IL-12 inversely regulates T-bet and eomesodermin expression during pathogen-induced CD8+ T cell differentiation.. J Immunol.

[37] Banerjee A, Gordon SM, Intlekofer AM, Paley MA, Mooney EC (2010). Cutting edge: The transcription factor eomesodermin enables CD8+ T cells to compete for the memory cell niche.. J Immunol.

[38] Kallies A, Xin A, Belz GT, Nutt SL (2009). Blimp-1 transcription factor is required for the differentiation of effector CD8(+) T cells and memory responses.. Immunity.

[39] Rutishauser RL, Martins GA, Kalachikov S, Chandele A, Parish IA (2009). Transcriptional repressor Blimp-1 promotes CD8(+) T cell terminal differentiation and represses the acquisition of central memory T cell properties.. Immunity.

[40] Ichii H, Sakamoto A, Hatano M, Okada S, Toyama H (2002). Role for Bcl-6 in the generation and maintenance of memory CD8+ T cells.. Nat Immunol.

[41] Ichii H, Sakamoto A, Kuroda Y, Tokuhisa T (2004). Bcl6 acts as an amplifier for the generation and proliferative capacity of central memory CD8+ T cells.. J Immunol.

[42] Ikeda H, Old LJ, Schreiber RD (2002). The roles of IFN gamma in protection against tumor development and cancer immunoediting.. Cytokine Growth Factor Rev.

[43] Blankenstein T, Qin Z (2003). The role of IFN-gamma in tumor transplantation immunity and inhibition of chemical carcinogenesis.. Curr Opin Immunol.

[44] Qin Z, Blankenstein T (2000). CD4+ T cell–mediated tumor rejection involves inhibition of angiogenesis that is dependent on IFN gamma receptor expression by nonhematopoietic cells.. Immunity.

[45] Dunn GP, Old LJ, Schreiber RD (2004). The immunobiology of cancer immunosurveillance and immunoediting.. Immunity.

[46] Qin Z, Schwartzkopff J, Pradera F, Kammertoens T, Seliger B (2003). A critical requirement of interferon gamma-mediated angiostasis for tumor rejection by CD8+ T cells.. Cancer Res.

[47] Barth RJ, Mule JJ, Spiess PJ, Rosenberg SA (1991). Interferon gamma and tumor necrosis factor have a role in tumor regressions mediated by murine CD8+ tumor-infiltrating lymphocytes.. J Exp Med.

[48] Prevost-Blondel A, Neuenhahn M, Rawiel M, Pircher H (2000). Differential requirement of perforin and IFN-gamma in CD8 T cell-mediated immune responses against B16.F10 melanoma cells expressing a viral antigen.. Eur J Immunol.

[49] Shankaran V, Ikeda H, Bruce AT, White JM, Swanson PE (2001). IFNgamma and lymphocytes prevent primary tumour development and shape tumour immunogenicity.. Nature.

[50] Tuttle TM, McCrady CW, Inge TH, Salour M, Bear HD (1993). gamma-Interferon plays a key role in T-cell-induced tumor regression.. Cancer Res.

[51] Seki N, Brooks AD, Carter CR, Back TC, Parsoneault EM (2002). Tumor-specific CTL kill murine renal cancer cells using both perforin and Fas ligand-mediated lysis in vitro, but cause tumor regression in vivo in the absence of perforin.. J Immunol.

[52] Meunier MC, Delisle JS, Bergeron J, Rineau V, Baron C (2005). T cells targeted against a single minor histocompatibility antigen can cure solid tumors.. Nat Med.

[53] Joshi NS, Kaech SM (2008). Effector CD8 T cell development: a balancing act between memory cell potential and terminal differentiation.. J Immunol.

[54] Vanden Bush TJ, Buchta CM, Claudio J, Bishop GA (2009). Cutting Edge: Importance of IL-6 and cooperation between innate and adaptive immune receptors in cellular vaccination with B lymphocytes.. J Immunol.

