# Supplementary figures and images for: CD40-Activated B Cells Can Efficiently Prime Antigen-Specific Naïve CD8+ T Cells to Generate Effector but Not Memory T cells
Source: PLoS One. 2012 Jan 23;7(1):e30139. doi: 10.1371/journal.pone.0030139 (PMC3264565; doi:10.1371/journal.pone.0030139)

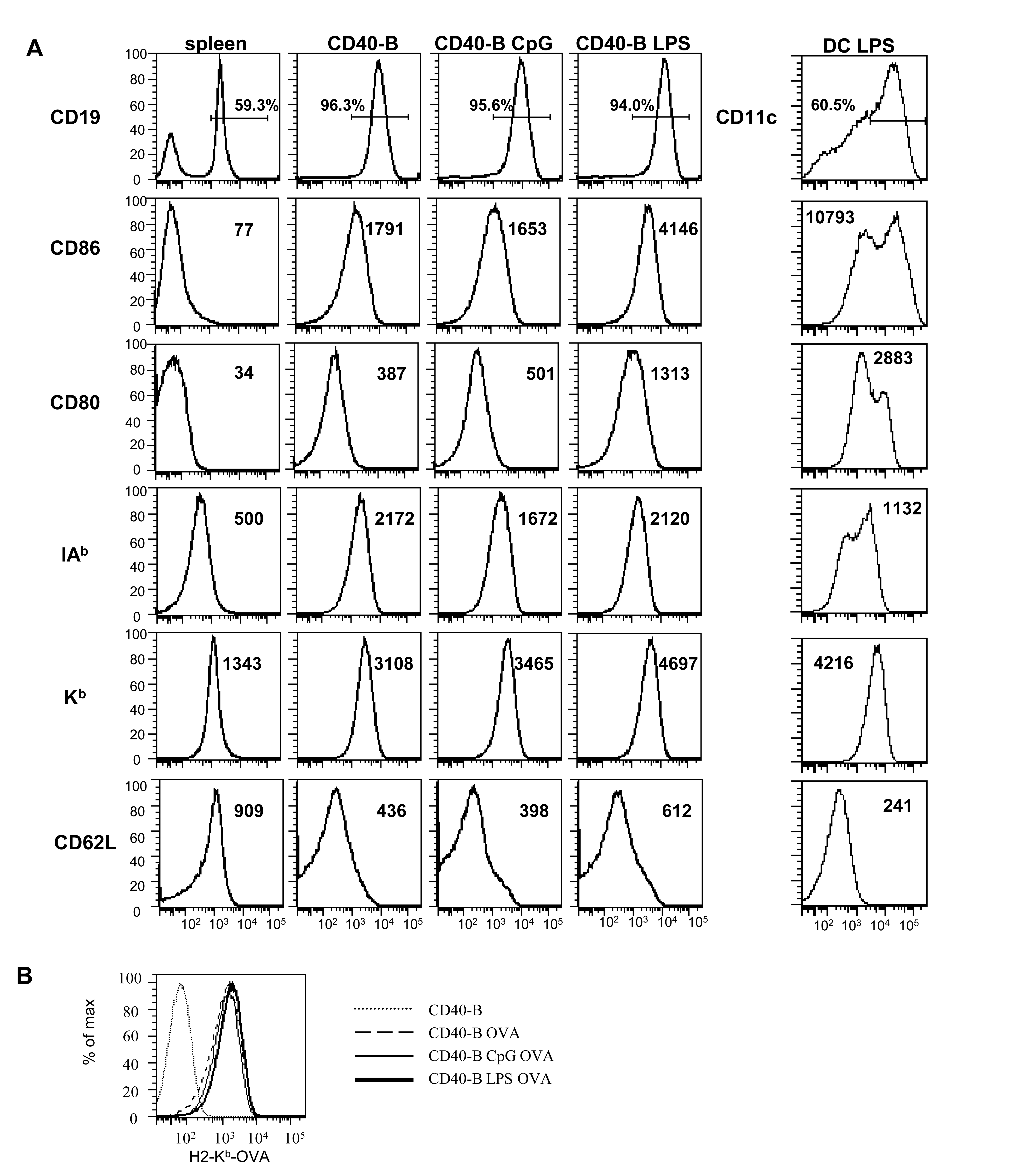

Supplement: Figure S1 — Characterization of CD40-B cells. A. Phenotype of CD40-B cells and DCs. B cells were grown from B6SJL splenocytes on irradiated murine NIH-3T3 fibroblasts transfected with the mouse CD40L cDNA. After 3 days of culture, B cells were matured with LPS (1 µg/mL) or CpG-DNA (2 mM) for 24 h. Freshly isolated splenocytes were also stained as controls. Dendritic cells (DC) were obtained by culturing bone marrow cells during 7 days with GM-CSF and IL-4 and were matured with LPS (1 µg/mL) on day 6. Percentage of CD19+ cells, percentage of CD11c+ cells or mean fluorescence intensity are indicated on each histogram. CD86, CD80, IAb, Kb, and CD62L histograms were gated on CD19+ cells for CD40-B cells and splenocytes or on CD11c+ cells for DCs. B. Similar loading of the OVA peptide for each B cell culture conditions. SIINFEKL (OVA) peptide (4 µg/ml) was added on day 3 of culture. The Kb-OVA complex was detected using a monoclonal antibody (25.D1.16) that specifically recognizes this peptide-MHC complex. The staining for Kb-OVA is shown for each culture conditions. (TIF) [file pone.0030139.s001.tif]

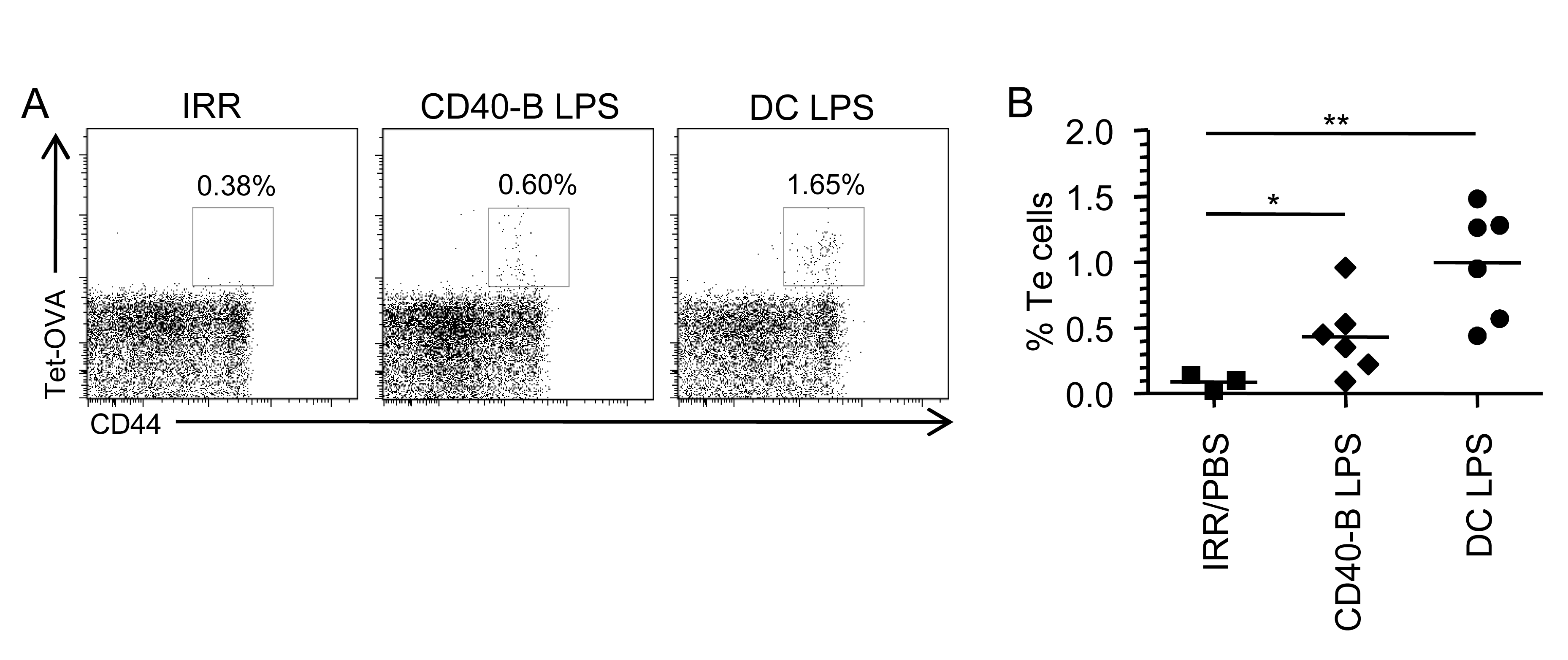

Supplement: Figure S2 — Immunization with CD40-B lymphocytes induces an endogenous response. C57BL/6 mice were immunized with 2×10^6^ CD40-B cells or DCs matured with LPS (1 µg/mL) and loaded with SIINFEKL (OVA) peptide. Responses were measured in the blood 7 days post-immunization with Kb-OVA tetramer staining. A. Representative dot plot are shown. Cells were previously gated on CD8+CD3+ cells. B. Percentage of response for each immunized mouse is shown. 2 independent experiments. Statistical analysis was done using a t-test. *p<0.05 and **p<0.01. (TIF) [file pone.0030139.s002.tif]

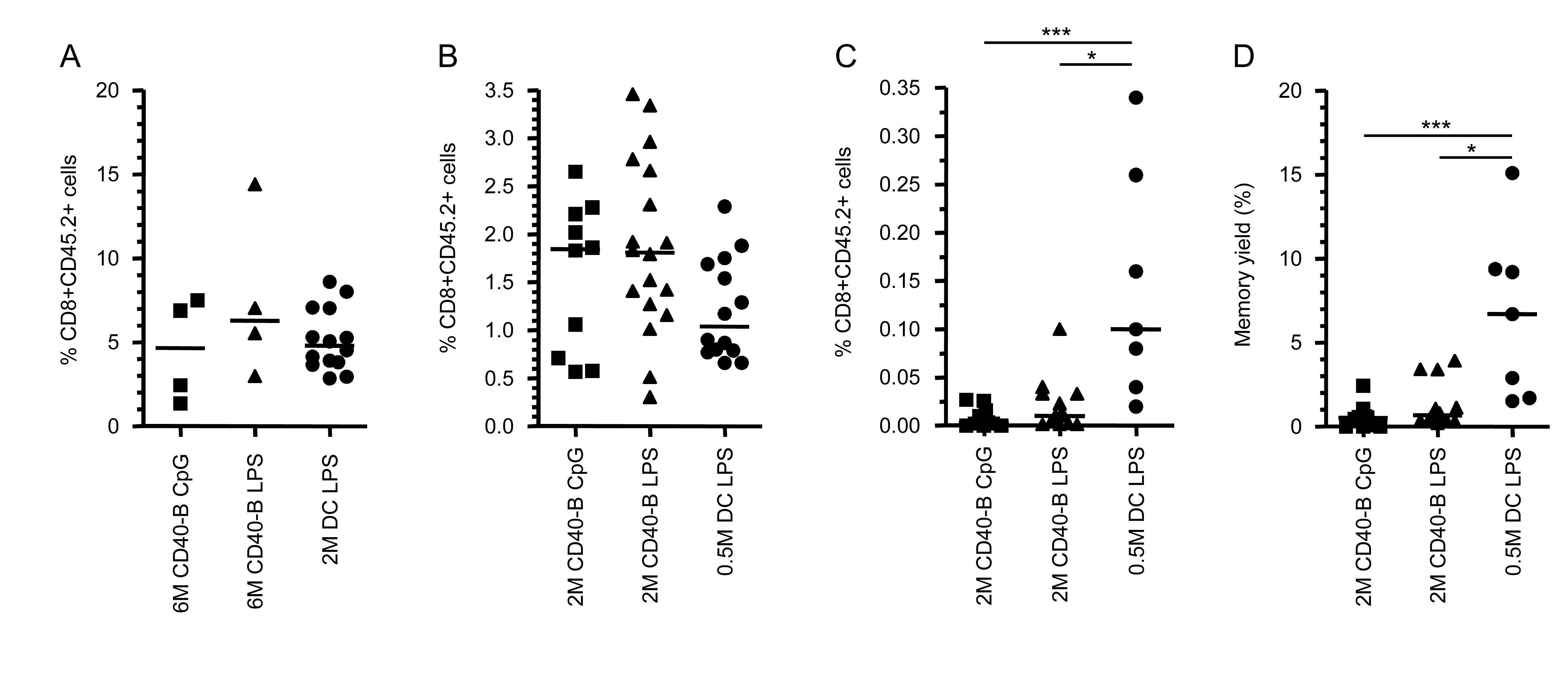

Supplement: Figure S3 — Immunization with CD40-B cells can generate an effector response comparable to immunization with DCs. 106 female OT-1 cells were adoptively transferred into B6SJL female mice followed by immunization with 2×10^6^ or 6×10^6^ CD40-B cells, matured with LPS (1 µg/mL) or CpG-DNA (2 mM) and loaded with 4 µg/mL of SIINFEKL (OVA) peptide. As a reference recipients were immunized with 0.5×10^6^ or 2×10^6^ DCs matured with LPS and loaded with OVA peptide. OVA-specific T cells (CD8+CD45.2+) effector response was analyzed in the same mouse by surgical removal of superficial lymph nodes at d4 and d30 or more. A. Percentage of Te cells (CD8+CD45.2+) generated with 6×10^6^ (6 M) CD40-B LPS OVA, 6 M CD40-B CpG OVA or 2×10^6^ (2 M) DC LPS OVA immunization. B. Percentage of Te cells (CD8+CD45.2+) obtained with 2 M CD40-B LPS OVA, 2 M CD40-B CpG OVA or 0.5×10^6^ (0.5 M) DC LPS OVA immunization. C. Percentage of Tm cells (CD8+CD45.2+) generated following immunization with 2 M CD40-C CpG OVA, 2 M CD40-B LPS OVA or 0.5 M DC LPS OVA. D. Memory yield (% of Tm cells generated from Te cells) is shown for the different immunization conditions (as in B). Each dot represents one mouse. Medians are shown and Kruskall-Wallis statistical analysis was performed. ** p<0.01, *** P<0.001. (TIF) [file pone.0030139.s003.tif]

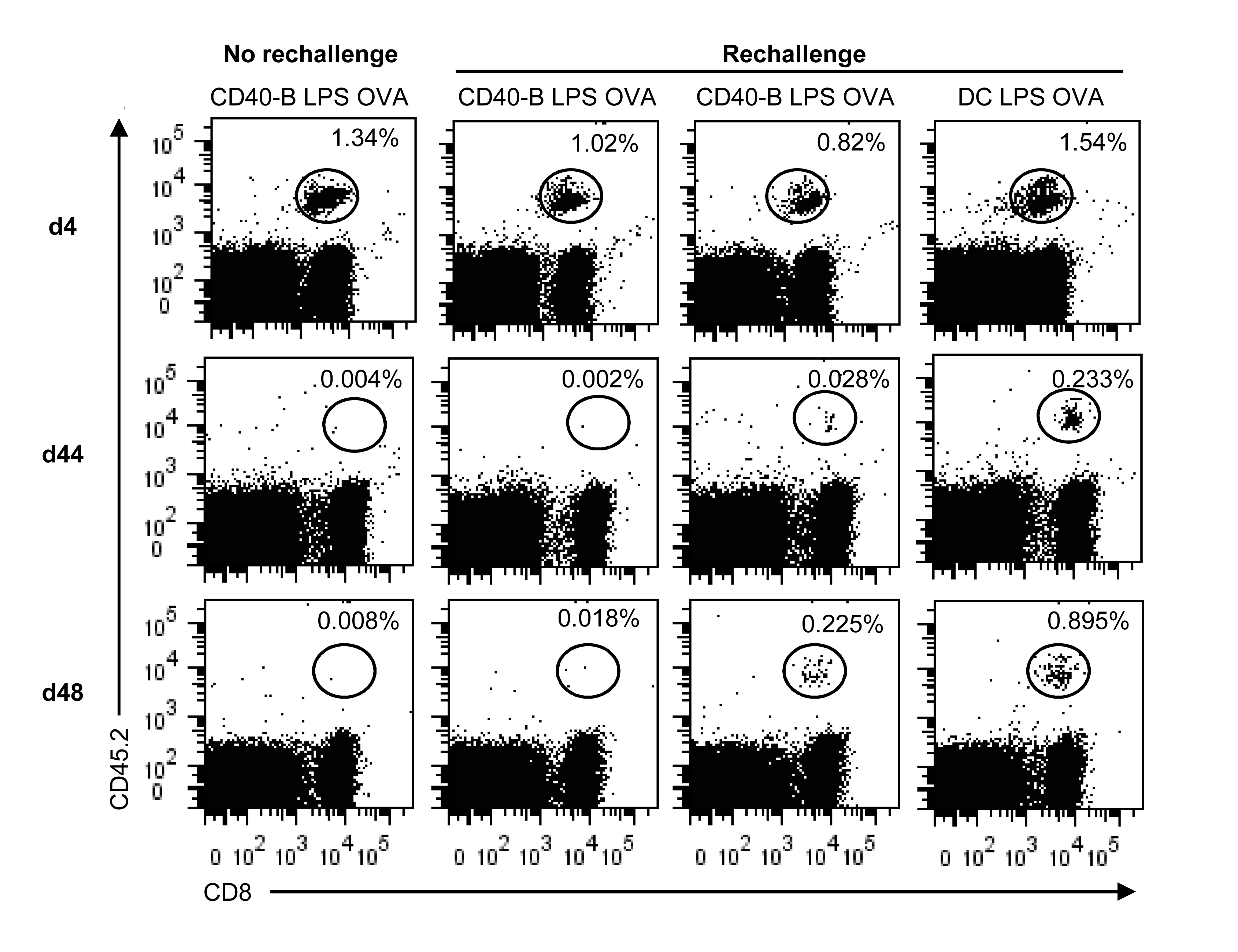

Supplement: Figure S4 — Recall response of CD8+ memory T cells after a challenge with Ag-pulsed DCs. 106 female OT-1 T cells (CD8+CD45.2+) were adoptively transferred into congenic B6SJL female mice (CD45.1+) followed by immunization two days later with 2×10^6^ CD40-B cells, matured or not with LPS (1 µg/mL) or CpG (2 mM) and loaded with 4 ug/mL of OVA peptide or with an irrelevant peptide (IRR). As a reference recipients were immunized with 2×10^6^ DCs matured with LPS and loaded with OVA peptide. The presence of OVA-specific T cells (CD8+CD45.2+) were analyzed in the same mouse by surgical removal of superficial lymph nodes at d4 (effector; top row) and d44 (memory; middle row) of the primary response. At d45 post-immunisation, mice were challenged or not with 1.25×10^6^ DCs matured with LPS and pulsed with the OVA peptide. Three days (d48) post-challenge, mice were sacrificed and OVA specific CD8+ memory T cell (CD8+CD45.2+) expansion was evaluated in the lymph nodes (bottom row). (TIF) [file pone.0030139.s004.tif]

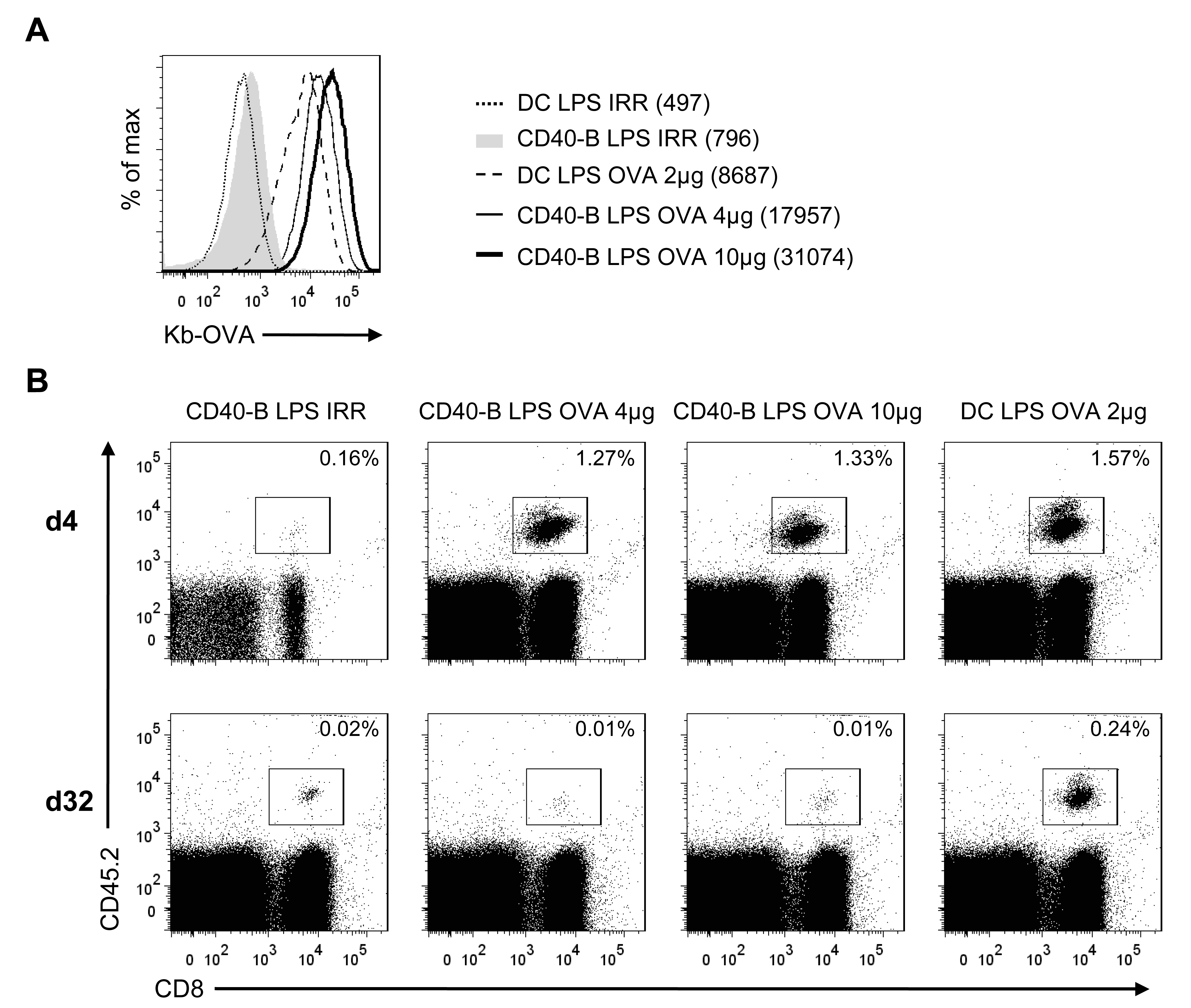

Supplement: Figure S5 — Defective memory T cell generation with CD40-B cell vaccination is not due to lower epitope density. A. CD40-B cells express a higher density of epitope than DCs. The level of Kb-OVA MHC-peptide complex expressed by the different APCs was evaluated using a monoclonal antibody (25.D1.16) that specifically recognizes this peptide-MHC complex. The staining for Kb-OVA is shown for each APC type used for immunization. The mean fluorescence intensity of Kb-OVA expression is indicated for each APC type next to the legend. B. Increasing epitope density on CD40-B cells does not improve the generation of CD8+ memory T cells. 106 female OT-1 T cells (CD8+CD45.2+) were adoptively transferred into congenic B6.SJL female mice (CD45.1+) followed by immunization two days later with 2×10^6^ CD40-B cells, matured with LPS (1 µg/mL) and loaded with different concentrations of OVA peptide (4 or 10 µg/ml) or with an irrelevant peptide (IRR). As a reference recipients were immunized with 2×10^6^ DCs matured with LPS and loaded with 2 µg/ml of OVA peptide. The dot plots show the effector (d4) and memory responses (d32) for each vaccination conditions. The percentage of effector and memory (CD8+CD45.2+) T cells generated are indicated on each dot plot. (TIF) [file pone.0030139.s005.tif]

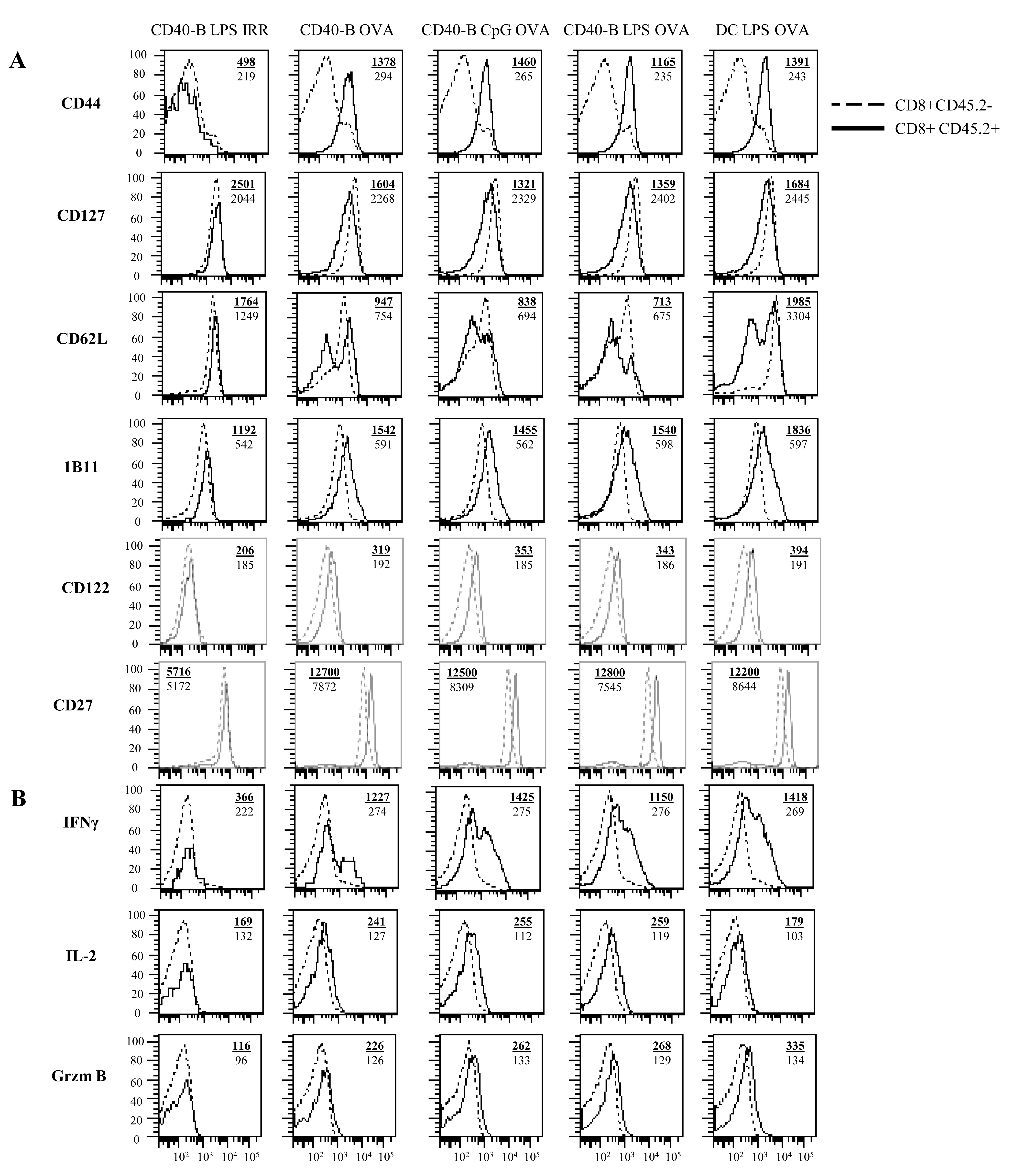

Supplement: Figure S6 — Phenotype and function of effector CD8+ T cells generated after CD40-B cell immunization. A. Phenotype of OVA-specific CD8+ effector T cells. 106 female OT-1 cells were adoptively transferred into B6SJL female mice followed by immunization with 2×10^6^ CD40-B cells, matured or not with LPS (1 µg/mL) or CpG-DNA (2 mM) and loaded with 4 µg/mL SIINFEKL (OVA) or an irrelevant peptide (IRR). As a reference recipients were immunized with 2×10^6^ DCs matured with LPS and loaded with OVA peptide. 4 days post-immunization, lymph nodes were removed by surgery and the phenotype of effectors was analysed by flow cytometry. The overlays show expression of the different cell surface markers by OVA-specific T cells (CD8+CD45.2+) compared to endogenous T cells (CD8+CD45.2−). The mean fluorescence intensity (MFI) is indicated on each histogram, the upper bold number indicates the MFI of OVA-specific effectors (CD8+CD45.2+) while the lower number is for the endogenous population (CD8+CD45.2−). B. Acquisition of effector functions by OVA-specific CD8+ T cells following CD40-B cell vaccination. OVA-specific effector CD8+ T cells were generated as in A. 4 days post-immunization, cells were restimulated for 4 h with PMA/ionomycin in the presence of brefeldin A to evaluate IFN-γ, IL-2 and granzyme B production. The overlays show production of the different effector molecules by OVA-specific T cells (CD8+CD45.2+) compared to endogenous T cells (CD8+CD45.2−). The MFI is indicated on each histogram, the upper bold number indicates the MFI of OVA-specific effectors (CD8+CD45.2+) while the lower number is for the endogenous population (CD8+CD45.2−). (TIF) [file pone.0030139.s006.tif]

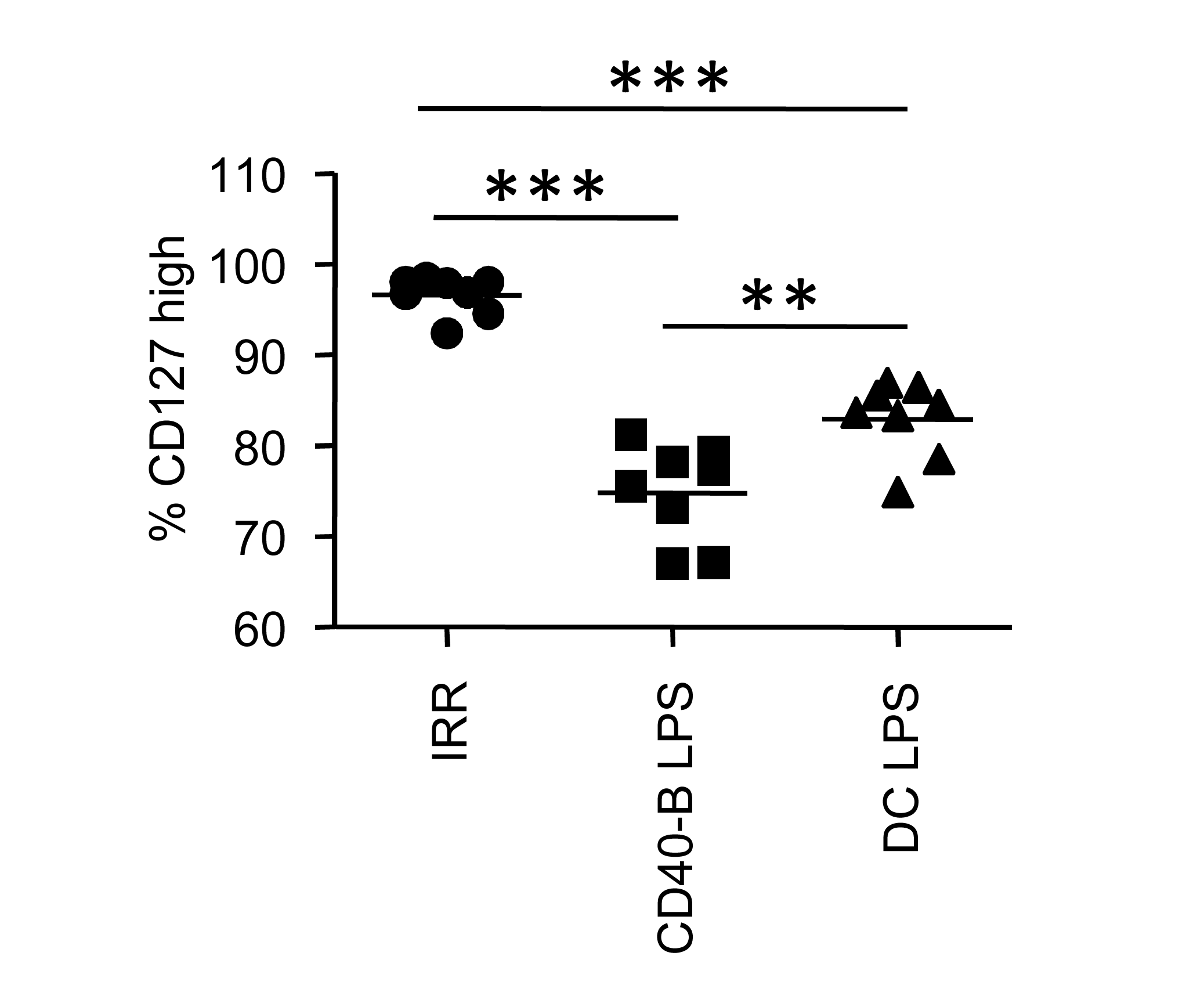

Supplement: Figure S7 — The majority of effectors generated with CD40-B cell immunization express high level of CD127. 106 female OT-1 cells were adoptively transferred into B6SJL female mice followed by immunization with 2×10^6^ CD40-B cells or DCs matured with LPS (1 µg/mL) and loaded with SIINFEKL (OVA). OVA-specific T cells (CD8+CD45.2+) effectors response was analyzed in superficial lymph nodes at d4 post-immunization. Percentage of effectors highly expressing CD127 are shown. 3 independent experiments, 2–3 mouse per experiments. (TIF) [file pone.0030139.s007.tif]

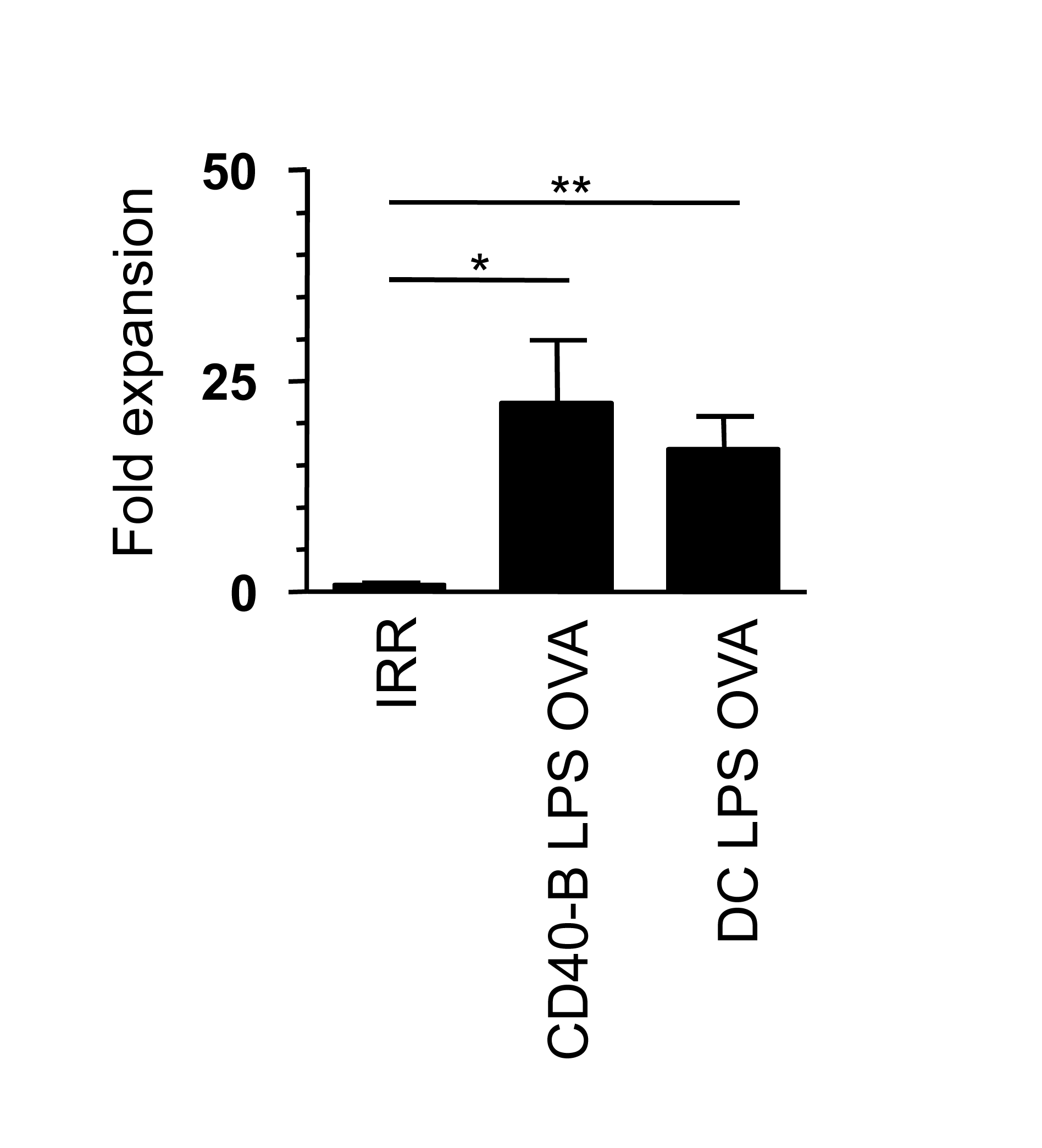

Supplement: Figure S8 — Similar CD8+ effector T cell expansion following Listeria monocytogenes challenge. 106 female OT-1 T cells (CD8+CD45.2+) were adoptively transferred into congenic B6SJL female mice (CD45.1+) followed by immunization two days later with 2×10^6^ CD40-B cells, matured or not with LPS (1 µg/mL) and loaded with 4 µg/mL OVA or with an irrelevant peptide (IRR). As a reference recipients were immunized with 5×10^5^ DCs matured with LPS and loaded with OVA peptide. Four days post-immunization, mice were challenged with a lethal dose of Lm-OVA (105 CFU). On the day of challenge and 3 d post-infection (peak of bacterial load) blood was harvested, stained and analyzed by flow cytometry. Percentage of CD8+CD45.2+ effectors before and after challenge were determined to calculate fold expansion. Mean +/− SEM are shown. 2–4 mice per conditions, 3 independent experiments. * p<0.05 and ** p<0.01. (TIF) [file pone.0030139.s008.tif]
